# Iron Deficiency and Human Brain Electrophysiology Across the Lifespan: A Systematic and Mechanistic Review of Resting EEG, Event-Related Potentials, and Treatment Responsiveness

**DOI:** 10.3390/nu18182955

**Published:** 2026-09-09

**Authors:** James Chmiel, Jolanta Góral-Półrola, Patrycja Leśnicka, Marta Kopańska

**Affiliations:** 1 Faculty of Physical Culture and Health, Institute of Physical Culture Sciences, University of Szczecin, Al. Piastów 40B Blok 6, 71-065 Szczecin, Poland; 2 Faculty of Pedagogy and Psychology, Jan Kochanowski University of Kielce, 25-029 Kielce, Poland; jolagoralpolrola@gmail.com; 3 Department of Medical Communication and Professional Competency Development, Faculty of Medicine, Collegium Medicum, University of Rzeszów, 35-959 Rzeszów, Poland; patrycjazagata@gmail.com

**Keywords:** iron deficiency, iron-deficiency anemia, electroencephalography, EEG, event-related potentials, P300, brain oscillations, neurodevelopment, cognitive function, iron supplementation

## Abstract

Introduction: Iron is essential for cerebral energy metabolism, neurotransmitter synthesis, myelination, and neural development. Iron deficiency may therefore impair brain function even before overt anemia develops. This systematic and mechanistic review synthesized EEG findings associated with iron deficiency across the lifespan and evaluated electrophysiological changes following iron treatment. Materials and Methods: PubMed/MEDLINE, Scopus, Web of Science, Embase, PsycINFO, and Google Scholar were searched from inception to July 2026. Human studies examining iron deficiency or iron-deficiency anemia using EEG, quantitative EEG, task-related oscillations, event-related potentials, or cortical-evoked potentials formed the core evidence base. Two additional studies of chronic kidney disease-related or mixed-etiology anemia were retained as contextual evidence to help distinguish iron-specific electrophysiological effects from abnormalities associated more generally with anemia severity and reduced oxygen-carrying capacity. Risk of bias was assessed using RoB 2 and ROBINS-I. Because of substantial heterogeneity, findings were synthesized narratively. Results: Thirty reports were retained for narrative synthesis, covering participants from the neonatal period to adulthood. Twenty-eight formed the core iron-specific evidence base, whereas two were analyzed separately as contextual anemia evidence. Iron deficiency was commonly associated with increased slow-wave activity, reduced alpha activity, delayed sensory and cognitive processing, prolonged P300 and N2 latencies, altered frontal alpha asymmetry, and impaired differentiation between relevant and irrelevant stimuli. Reductions in P300 amplitude were less consistent but were observed in severe anemia, demanding cognitive tasks, and after infantile iron-deficiency anemia. Iron supplementation improved several spectral and ERP outcomes, although recovery was variable and some abnormalities persisted after hematological correction. Interpretation was limited by heterogeneous diagnostic criteria, small samples, inconsistent EEG methods, residual confounding, variable risk of bias, and the partial non-independence of reports arising from overlapping longitudinal cohorts or parent trials. Findings from the contextual anemia studies were not interpreted as direct evidence of an effect of iron deficiency. Conclusions: Iron deficiency may disrupt the timing, synchronization, and maturation of neural activity through impaired energy metabolism, neurotransmission, myelination, and synaptic development. EEG may be useful for detecting functional brain alterations and monitoring treatment response, but no specific electrophysiological biomarker can currently be established.

## 1. Introduction

Iron deficiency is the most prevalent micronutrient deficiency worldwide and remains an important public-health problem across both low- and high-income countries. It ranges from depleted iron stores and iron-restricted erythropoiesis to iron-deficiency anemia (IDA), when iron availability becomes insufficient for normal hemoglobin synthesis [1,2,3]. The main causes include inadequate dietary intake, increased physiological requirements during periods of growth or pregnancy, impaired gastrointestinal absorption, and chronic blood loss. Functional iron deficiency may also occur when inflammation and hepcidin-mediated sequestration restrict the mobilization and utilization of stored iron [1,2,3,4,5]. The condition is particularly common during infancy, childhood, adolescence, pregnancy, and in menstruating women because of increased physiological requirements or recurrent blood loss [4,5]. The main causes of iron deficiency across the lifespan are presented in Table 1.

The consequences of iron deficiency extend beyond impaired hemoglobin synthesis and oxygen transport. Iron is essential for mitochondrial energy metabolism, neurotransmitter synthesis, myelination, and normal neuronal development [6,7,8,9,10,11]. Iron-dependent processes contribute to cellular energy production, synaptic transmission, neuronal plasticity, dopaminergic function, and the maturation of oligodendrocytes and white matter [6,7,8]. These functions are especially important during fetal development, infancy, and early childhood, when neurogenesis, synaptogenesis, hippocampal maturation, and myelination are particularly active. Consequently, prolonged or severe iron deficiency during sensitive developmental periods may be associated with persistent alterations in neural function even after systemic iron status has improved [7,8,9,10,11].

Iron deficiency has also been associated with impairments in attention, learning, memory, psychomotor development, language, and broader cognitive functioning, although the magnitude and persistence of these effects vary according to age, severity and duration of deficiency, nutritional and socioeconomic factors, and study design [12,13,14,15,16]. Prospective and intervention studies suggest that the improvement in iron status may benefit attention, memory, learning efficiency, and processing speed in some iron-deficient populations [17,18]. Maternal iron-deficiency anemia has additionally been associated with alterations in maternal cognitive and affective functioning and mother–infant interaction [19]. These findings suggest that the effects of iron deficiency on the nervous system may involve both reversible functional disturbances and, particularly when deficiency occurs during critical developmental periods, more persistent alterations.

Electroencephalography (EEG) provides a non-invasive measure of cerebral electrical activity with high temporal resolution and can be used across the lifespan [20,21,22,23,24]. Resting-state EEG and quantitative EEG (qEEG) allow the assessment of spontaneous oscillatory activity, including spectral power and slowing, whereas task-related EEG and event-related potentials (ERPs) provide information about sensory, attentional, memory, and higher-order cognitive processing [21,25,26,27,28,29,30]. Electrophysiological measures may therefore detect functional alterations that are not necessarily captured by conventional clinical or behavioral assessments. Changes in spectral activity, oscillatory organization, or ERP amplitude and latency may provide complementary information about the timing, efficiency, and organization of neural processing.

Despite extensive research on the cognitive, developmental, and behavioral consequences of iron deficiency, its effects on human brain electrophysiology have not been comprehensively synthesized across the lifespan. Available studies include neonatal, infant, childhood, adolescent, and adult populations and use heterogeneous approaches, including conventional EEG, resting-state qEEG, task-related oscillations, sensory-evoked potentials, and cognitive ERPs. Differences in the definition and severity of iron deficiency, presence of anemia, developmental stage, EEG methodology, and cognitive paradigms make it difficult to determine which electrophysiological findings are reproducible and which may be specific to particular populations or experimental conditions. Furthermore, it remains unclear whether electrophysiological abnormalities improve following iron treatment and whether some alterations may persist after hematological recovery.

Therefore, the aim of this systematic review was to synthesize human evidence on electrophysiological alterations associated with iron deficiency across the lifespan, including resting and quantitative EEG, task-related oscillatory activity, sensory-evoked potentials, and cognitive ERPs. We additionally examined changes following iron supplementation or the correction of iron deficiency and considered the methodological quality and risk of bias of the available evidence. Particular attention was given to the consistency of electrophysiological findings across developmental stages, the distinction between iron-specific effects and abnormalities potentially related to anemia, and the extent to which electrophysiological measures may have potential value for detecting or monitoring functional brain alterations associated with iron deficiency.

## 2. Materials and Methods

### 2.1. Review Design and Reporting Framework

This systematic review was designed to identify, evaluate, and synthesize human studies examining the relationship between iron deficiency and cerebral electrophysiological activity measured using electroencephalography. The review covered electrophysiological alterations associated with iron deficiency, iron depletion, and iron-deficiency anemia across different stages of life, from the neonatal period to adulthood. Particular attention was given to resting-state and conventional EEG abnormalities, quantitative EEG measures, task-related oscillatory activity, event-related potentials, and changes in electrophysiological outcomes following iron supplementation or the correction of iron deficiency.

The review was conducted and reported in accordance with the Preferred Reporting Items for Systematic Reviews and Meta-Analyses 2020 guidelines. Eligibility criteria, search concepts, data-extraction categories, risk-of-bias procedures, and the approach to evidence synthesis were defined before the final synthesis was undertaken. The study-selection process was documented using a PRISMA flow diagram.

### 2.2. Eligibility Criteria

Studies were eligible for the core synthesis if they (1) included human participants; (2) evaluated iron deficiency, iron-deficiency anemia, or iron-status biomarkers; (3) assessed cerebral electrophysiology using scalp EEG, quantitative EEG, event-related potentials, or cortical-evoked potentials; and (4) reported an interpretable electrophysiological outcome related to iron status or iron treatment. Eligible designs included observational studies and randomized or non-randomized intervention studies. Non-human studies, non-original publications, case reports, conference-only publications, studies without cortical EEG-derived outcomes, and studies in which iron-related exposure could not be established were excluded.

Iron deficiency could be defined using serum ferritin, serum iron, transferrin saturation, soluble transferrin receptor, total iron-binding capacity, body iron, zinc protoporphyrin-to-heme ratio, mean corpuscular volume, mean corpuscular hemoglobin, or a combination of hematological and biochemical indicators. Evidence was classified according to the specificity of the iron-status definition. Studies using biochemical indicators to confirm iron deficiency or iron-deficiency anemia were classified as confirmed iron-specific evidence. Studies using hemoglobin-based anemia classifications were retained as probable iron-related evidence when the original investigators identified iron deficiency as the principal or likely cause of anemia, even when ferritin or other biochemical indicators were unavailable. The absence of biochemical confirmation was documented during data extraction, considered in the risk-of-bias assessment, and reflected in the interpretive weight assigned to the findings.

Two additional reports that did not meet the criteria for the core iron-specific synthesis were retained as contextual anemia evidence. One examined severe anemia associated with chronic kidney disease, whereas the other included a mixed-anemia population in which iron-deficiency anemia accounted for a substantial proportion of cases but subgroup-specific EEG findings were unavailable. These reports were retained because they provided relevant information about the association between anemia severity, hemoglobin concentration, cerebral oxygen limitation, and electrophysiological abnormalities. They were not treated as evidence that the reported EEG changes were caused specifically by iron deficiency.

Eligible study designs included randomized controlled trials, non-randomized supplementation studies, prospective and retrospective cohort studies, case–control studies, cross-sectional comparisons, longitudinal developmental studies, and correlational studies investigating associations between iron biomarkers and EEG measures. Intervention studies were included when they assessed electrophysiological changes following oral iron, iron-fortified foods, iron-containing micronutrient preparations, or another intervention intended to improve iron status. Studies were not required to include a healthy control group when they reported longitudinal EEG changes following treatment or associations between iron biomarkers and electrophysiological outcomes.

Eligible EEG outcomes included absolute and relative spectral power in the delta, theta, alpha, beta, and gamma frequency ranges; peak frequency and oscillatory power; aperiodic spectral parameters; frontal alpha asymmetry; EEG slowing; background organization; paroxysmal or other conventional EEG abnormalities; task-related oscillatory responses; and ERP amplitude, latency, morphology, topography, or condition differentiation. Cortically generated auditory and visual evoked responses were included when they were derived from scalp EEG. Behavioral and cognitive findings were extracted as secondary outcomes when they were reported alongside the electrophysiological results.

Studies were excluded when they (1) were conducted exclusively in animals or in vitro models; (2) did not include participants with confirmed or probable iron deficiency and did not provide prespecified contextual evidence concerning the cerebral electrophysiological effects of anemia severity; (3) did not report an EEG-derived outcome; (4) examined only peripheral nerve conduction, auditory brainstem responses, or other brainstem parameters without a cortical EEG or ERP measure; (5) were reviews, meta-analyses, editorials, letters, protocols, conference papers, conference abstracts, posters, or case reports; or (6) did not provide sufficient methodological or outcome information for interpretation. Studies involving anemia of mixed, uncertain, or clearly non-iron etiology were excluded from the core iron-specific synthesis when the effects of iron deficiency could not be distinguished or reasonably inferred. However, such reports could be retained in a separate contextual category when they provided directly relevant cortical EEG or ERP evidence concerning anemia severity or reduced oxygen-carrying capacity. Contextual studies were clearly identified throughout the synthesis and were not used to support iron-specific causal conclusions.

### 2.3. Information Sources and Search Strategy

A systematic literature search was conducted in PubMed/MEDLINE, Scopus, Web of Science Core Collection, Embase, PsycINFO, and Google Scholar. Searches covered each database from inception to the final search date. The database-specific searches were performed on 1 July 2026. No restrictions were applied regarding participant age, sex, geographical location, or year of publication. Results were restricted to publications in English.

The search strategy combined terms describing iron deficiency or iron-deficiency anemia with terms describing EEG and cerebral electrophysiology. Because database interfaces differ in their controlled vocabulary, field codes, phrase searching, and Boolean syntax, the strategy was translated separately for each database rather than applying a single identical search string. The complete reproducible search strategy for each information source, including the exact query as entered, database or platform, date of search, limits applied, and number of records retrieved, is provided in Supplementary Table S1.

The search concepts included terms related to iron deficiency (“iron deficiency”, “iron deficient”, “iron depletion”, “depleted iron stores”, “low iron”, “low ferritin”, “iron-deficiency anemia”, “iron deficiency anemia”, and IDA) and electrophysiology (EEG, electroencephalography, electroencephalogram, qEEG, “quantitative EEG”, “brain electrical activity”, “event-related potential”, ERP, “evoked potential”, P300, P3, P1, N1, N100, P2, P200, N2, N200, “slow wave”, FN400, “spectral power”, “brain oscillations”, and “alpha asymmetry”). Search syntax and field specifications were adapted to the requirements of each database; the exact executed strings are reproduced verbatim in Supplementary Table S1.

Google Scholar was used as a supplementary search source rather than as the sole basis for the systematic identification of records. The Google Scholar search was conducted on 1 July 2026 using the following prespecified queries: “iron deficiency” EEG; “iron deficiency anemia” EEG; “iron deficiency” “event-related potential”; “iron deficiency anemia” “event-related potential”; “iron deficiency” P300; “low ferritin” EEG. Results were screened in the order returned by Google Scholar. Duplicate records and records already identified through the bibliographic databases were removed before eligibility assessment. The number of Google Scholar records screened, the query or queries used, and the resulting number of potentially eligible records are reported in Supplementary Table S1.

In addition to electronic database searching, the reference lists of all eligible articles and relevant reviews were examined manually. Forward-citation searching of eligible studies was also performed, together with database functions identifying similar or related articles where available. Records identified through these supplementary methods were subjected to the same eligibility criteria as database-derived records.

All retrieved references were imported into reference-management software. Duplicate records were identified electronically and subsequently checked manually before title and abstract screening.

### 2.4. Study Selection

Study selection was conducted in two stages. First, titles and abstracts were screened against the predefined eligibility criteria. Records were excluded at this stage when they clearly did not investigate iron deficiency, did not use EEG or a related cortical electrophysiological method, involved non-human models, or represented non-original publications.

The full texts of potentially relevant articles were then retrieved and evaluated. During full-text screening, particular attention was paid to the method used to define iron status, the etiology of anemia, the type and anatomical level of the electrophysiological measurement, the presence of original empirical data, and the availability of interpretable EEG results. Studies assessing only auditory brainstem or other subcortical responses were distinguished from studies measuring cortical auditory or visual potentials. During full-text assessment, reports were additionally classified as core iron-specific evidence, probable iron-related evidence, or contextual anemia evidence. Contextual classification was used when iron-specific EEG results could not be isolated but the study contributed directly relevant information concerning anemia severity and cortical electrophysiology.

**Figure 1:**
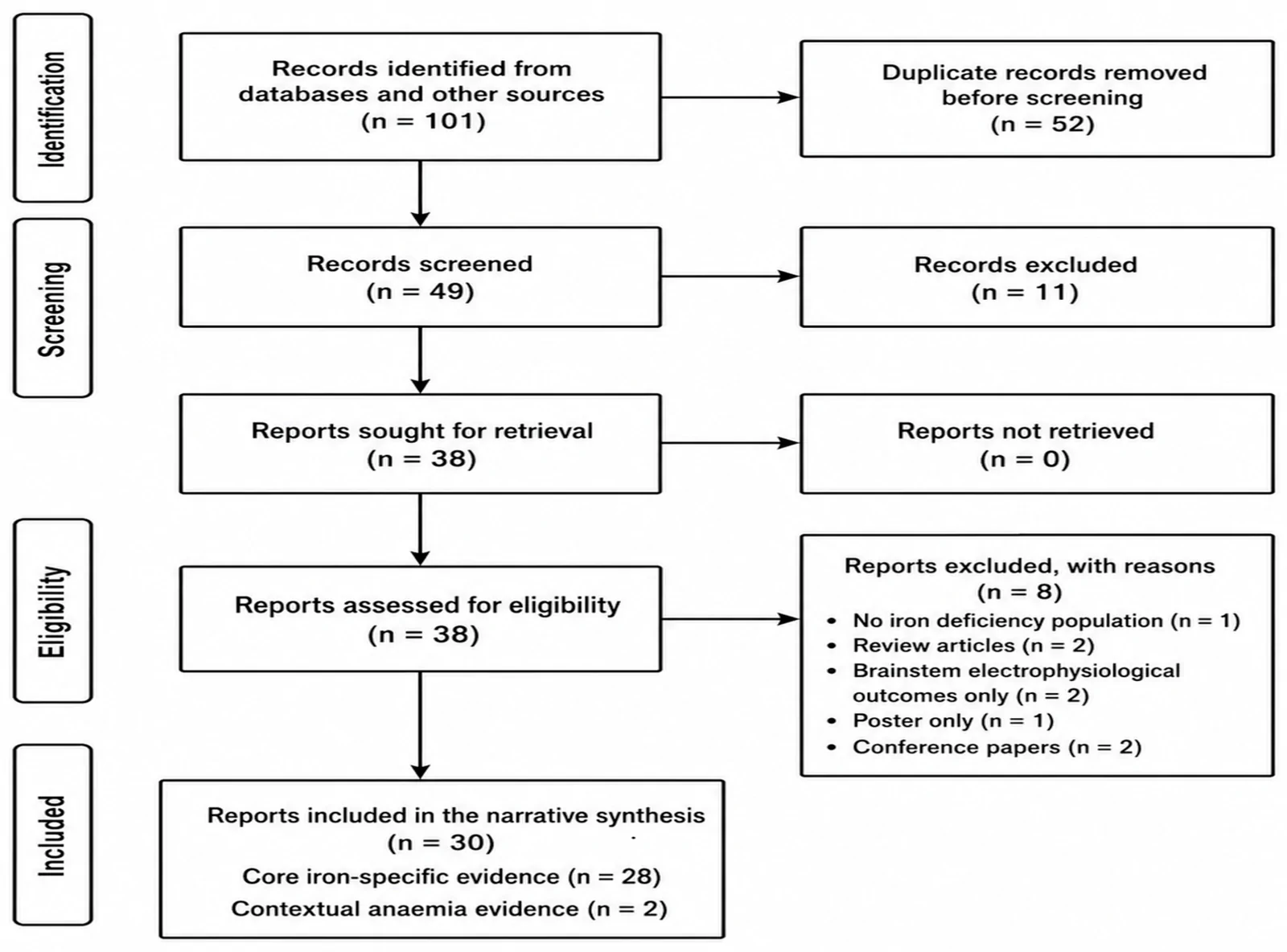
PRISMA 2020 flow diagram of study identification, screening, eligibility assessment, and inclusion in the systematic review.

Screening was performed independently by two reviewers. After removal of duplicate records, titles and abstracts of all unique records were screened against the predefined eligibility criteria. Records that clearly failed to meet the inclusion criteria were excluded at this stage. Full-text reports were then sought for all records considered potentially eligible. Reports that could not be retrieved, if any, were recorded separately. All retrieved full-text reports were independently assessed against the eligibility criteria, and a specific reason for exclusion was recorded for each report excluded at this stage. Disagreements at either screening stage were resolved through discussion and re-examination of the relevant record or report; when consensus could not be reached, a third reviewer was consulted. Consistent with PRISMA 2020 terminology, “records” refers to database or other-source citations identified before full-text retrieval, whereas “reports” refers to retrieved full-text publications. The numbers of records identified, records removed before screening, records screened, records excluded, reports sought for retrieval, reports not retrieved, reports assessed for eligibility, reports excluded with reasons, and studies included in the review are presented in the PRISMA 2020 flow diagram (Figure 1).

### 2.5. Data Extraction

Data were extracted using a standardized extraction form developed for this review. The following information was collected from each included study:

1. General study characteristics: First author, publication year, country, recruitment setting, and study design.
2. Participant characteristics: Total sample size, number of participants in each group, age or developmental stage, sex distribution, health status, relevant clinical characteristics, and inclusion and exclusion criteria.
3. Iron-status assessment: Biomarkers used to determine iron status, diagnostic thresholds, definition of iron deficiency or iron-deficiency anemia, severity of deficiency, presence or absence of anemia, inflammatory markers where available, and timing of biological sampling relative to EEG assessment.
4. Intervention characteristics: Form and dose of iron, route of administration, duration of supplementation, adherence, placebo or comparison condition, co-administered micronutrients, timing of post-treatment EEG assessment, and duration of follow-up.
5. EEG acquisition and preprocessing: Recording condition, behavioral state, task paradigm, number and location of electrodes, reference montage, sampling rate, filtering, artifact-rejection procedures, epoch definition, frequency-band definitions, and analytical software or procedures where reported.
6. Electrophysiological outcomes: Resting or task-related spectral power, ERP components, peak amplitude and latency, waveform morphology, scalp distribution, stimulus or condition differentiation, frontal asymmetry, conventional EEG abnormalities, background slowing, oscillatory peak characteristics, aperiodic activity, and other reported EEG indices.
7. Behavioral and cognitive outcomes: Task accuracy, reaction time, attention, memory, executive functioning, learning, intelligence, developmental performance, emotional behavior, and other outcomes assessed alongside EEG.
8. Associations and treatment effects: Relationships between EEG outcomes and ferritin, hemoglobin or other iron indicators; differences between iron-deficient and iron-sufficient participants; within-participant changes following treatment; persistence of abnormalities after correction of iron status; and evidence of statistical mediation between iron status, EEG activity, and cognitive performance.
9. Methodological information: Attrition, missing EEG data, artifact-related exclusions, statistical adjustments, correction for multiple comparisons, funding sources, reported conflicts of interest, and limitations relevant to interpretation.
10. Evidence classification: Classification as confirmed iron-specific evidence, probable iron-related evidence, or contextual anemia evidence; justification for the classification; availability of biochemical confirmation of iron deficiency; presence of mixed anemia etiologies; and availability of etiology-specific electrophysiological results.

For studies classified as contextual anemia evidence, particular attention was given to the etiology of anemia, ferritin concentrations, inflammatory or renal disease, treatment modality, and whether EEG findings were reported separately for participants with iron-deficiency anemia.

Potential cohort overlap was assessed using recruitment setting, enrollment period, participant characteristics, baseline and follow-up ages, intervention allocation, and descriptions of parent cohorts or trials in the original publications. Reports derived from the same or partially overlapping parent cohort were assigned a common cohort-overlap identifier in Table 2. Separate publications were retained when they addressed distinct developmental time points, electrophysiological paradigms, outcomes, or follow-up periods. However, reports originating from the same parent cohort were not treated as independent replications, even when the samples analyzed differed because of attrition, EEG-data usability, age-specific follow-up, or paradigm-specific inclusion criteria. Agreement between such reports was interpreted as within-cohort convergence rather than independent replication.

### 2.6. Outcomes

The primary outcome was the association between iron status and human cerebral electrophysiological activity. This included differences between iron-deficient and iron-sufficient participants, associations between continuous iron biomarkers and EEG measures, and changes in EEG outcomes following the improvement in iron status.

For synthesis, electrophysiological outcomes were organized into four broad categories: (1) resting and conventional EEG, (2) spectral and task-related oscillatory activity, (3) ERP and cortical evoked-potential measures, and (4) electrophysiological responses to iron treatment. The more specific outcomes listed below were treated as subdomains within these categories rather than as separate eligibility requirements.

The principal outcome domains were

1. Resting-state EEG spectral activity;
2. Conventional EEG abnormalities and background organization;
3. Frontal alpha asymmetry;
4. Task-related alpha, theta, beta, and gamma activity;
5. Early sensory and attentional ERP components, including P1, N1/N100, P2/P200, and N2/N200;
6. Later cognitive ERP components, particularly P3/P300;
7. Recognition-memory and late slow-wave responses;
8. Habituation, novelty detection, stimulus discrimination, and auditory or visual recognition responses; and
9. Electrophysiological normalization, partial recovery, or persistence following iron supplementation.

A secondary contextual outcome was the relationship between anemia severity or hemoglobin concentration and cerebral electrophysiological activity in studies in which iron deficiency was not confirmed or could not be isolated. This outcome was used only to evaluate the plausibility of an additional anemia-dependent mechanism involving reduced oxygen-carrying capacity. It was not used to infer an iron-specific effect.

Secondary outcomes included cognitive, behavioral, developmental, emotional, and performance measures reported in relation to EEG findings. These outcomes were used to determine whether electrophysiological differences were accompanied by measurable functional impairment or improvement.

### 2.7. Risk-of-Bias Assessment

Risk of bias was assessed using tools appropriate to the design of each study. Randomized controlled trials were evaluated using the Cochrane Risk of Bias 2 tool. The assessed domains included bias arising from the randomization process, deviations from intended interventions, missing outcome data, outcome measurement, and the selection of the reported result. Trials were classified as having low risk of bias, some concerns, or high risk of bias.

Non-randomized intervention studies and observational studies were evaluated using the Risk of Bias in Non-randomized Studies of Interventions tool (ROBINS-I). Because ROBINS-I was originally developed for non-randomized intervention studies, its domains were operationally adapted for purely observational exposure studies in which naturally occurring iron status, rather than an investigator-assigned intervention, constituted the exposure of interest. This adaptation was used as a structured domain-based assessment of the validity of the iron-status–EEG association and was not intended to imply that iron deficiency represented an intervention. For observational studies, the target exposure was defined as iron deficiency, iron-deficiency anemia, or the continuous iron-status measure assessed in the original study. Bias due to confounding and participant selection was evaluated in the usual manner. The ROBINS-I classification-of-intervention domain was reframed as classification or measurement of iron-status exposure and considered the biochemical specificity of the iron definition, the validity and timing of ferritin, serum iron, transferrin saturation, hemoglobin, erythrocyte indices, or other biomarkers, and the likelihood of misclassification between iron-deficient and iron-sufficient participants. The domain concerning deviations from intended interventions was interpreted as deviations from, or changes in, the defined exposure before electrophysiological assessment, including iron supplementation, transfusion, treatment of anemia, or other clinical management capable of modifying iron or hemoglobin status. When no meaningful post-classification deviation from exposure could occur, this domain was considered not applicable or low risk. The remaining domains—missing data, measurement of electrophysiological outcomes, and selection of the reported result—were applied directly. This approach allowed the same broad risk-of-bias framework to be used across heterogeneous non-randomized designs while directing the assessment toward threats to the validity of exposure–outcome associations.

Particular attention was paid to potential confounding by age, sex, socioeconomic status, nutritional status, inflammation, anemia severity, comorbid nutritional deficiencies, developmental risk factors, medication use, neurological disorders, and the underlying cause of iron deficiency. EEG-specific concerns included inadequate control of behavioral state, unblinded outcome assessment, small numbers of artifact-free trials, high attrition caused by unusable EEG recordings, inconsistent preprocessing, non-standard frequency-band definitions, selective electrode reporting, and insufficient correction for multiple EEG comparisons.

Risk-of-bias judgments were made independently by two reviewers, with disagreements resolved through discussion. Studies were not excluded solely because of their risk-of-bias classification; instead, risk of bias was incorporated directly into the interpretive weighting of the narrative synthesis. No numerical quality score or formal statistical weighting was applied. Findings from studies with fewer and less severe methodological concerns, biochemical confirmation of iron deficiency, appropriate comparison groups, or randomized designs were assigned greater interpretive weight. Findings from studies with serious ROBINS-I concerns were retained but treated as supportive rather than definitive evidence and were given greater weight only when corroborated by independent studies or by evidence with stronger exposure classification or study design. Findings from studies with a critical ROBINS-I judgment were not permitted to establish an iron-specific or causal conclusion on their own and were treated as low-confidence, hypothesis-generating, or contextual evidence. Similarly, findings from randomized trials judged at high risk of bias were down-weighted relative to more methodologically robust randomized evidence. When a serious- or critical-risk study was the sole source of a particular finding, that finding was explicitly described as preliminary or uncertain and did not determine the domain-level conclusion. When results differed across studies, greater interpretive weight was assigned to findings from studies with lower risk of bias and stronger iron-status ascertainment.

### 2.8. Data Synthesis

Because of substantial clinical and methodological heterogeneity, a quantitative meta-analysis was not considered appropriate. The included studies differed in participant age, developmental stage, severity and duration of iron deficiency, presence of anemia, diagnostic thresholds, study design, supplementation protocol, EEG recording condition, electrode montage, preprocessing procedures, frequency-band definitions, ERP paradigms, outcome quantification, and statistical reporting.

A structured narrative synthesis was therefore performed. Findings were first summarized according to participant characteristics, study design, iron-status definition, and EEG paradigm. Electrophysiological results were then grouped into conceptually related domains, including resting and conventional EEG, spectral slowing, alpha-band activity and frontal asymmetry, task-related oscillations, early sensory and attentional ERP components, P3/P300 and later cognitive processing, recognition-memory responses, auditory habituation, and other conventional EEG abnormalities.

Treatment studies were examined separately to determine whether electrophysiological abnormalities improved after the correction of iron status. Evidence of complete normalization, partial normalization, persistent abnormalities, and delayed treatment effects was distinguished. Developmental timing was considered when comparing prenatal or neonatal iron deficiency, postnatal deficiency, childhood deficiency, and iron depletion in adults.

The evidence was synthesized hierarchically according to the specificity of the iron-status assessment. Risk of bias constituted an additional level of this hierarchy. The synthesis therefore did not use simple vote counting in which every study contributed equally to a recurring pattern. A finding reported by a study with serious risk of bias contributed less to the overall interpretation than a comparable finding supported by a better-controlled or randomized study, and a finding from a study with critical risk of bias was not counted as independent evidence sufficient to establish an iron-specific association or treatment effect. Critical-risk findings could strengthen an interpretation only when they were directionally consistent with findings from methodologically stronger studies; otherwise, they were retained descriptively but did not alter the principal conclusion for that electrophysiological domain. The greatest interpretive weight was assigned to findings supported by biochemical confirmation of iron deficiency, replicated across independent samples, accompanied by consistent cognitive or behavioral effects, associated with the severity of iron depletion, or responsive to iron treatment. Studies in which iron-deficiency anemia was identified by the original investigators but defined only by hemoglobin or incomplete hematological criteria were retained as probable iron-related evidence and interpreted with reduced confidence. Studies involving chronic kidney disease-related or mixed-etiology anemia were presented separately as contextual evidence. Their findings were used to examine anemia-related oxygen limitation and severity gradients but were not counted as independent support for an iron-specific electrophysiological effect. Findings based on small samples, uncorrected multiple comparisons, high attrition, or isolated electrode effects were also interpreted cautiously. Conclusions regarding iron deficiency were based principally on the core iron-specific evidence base.

When evaluating the recurrence, consistency, or replication of electrophysiological findings, the unit of independence was the parent cohort rather than the individual publication. Multiple reports derived from an overlapping cohort could provide evidence of persistence across developmental time points or convergence across different electrophysiological paradigms, but they did not increase the number of independent replications. Accordingly, terms such as “replicated,” “consistent across independent studies,” or “recurrent across cohorts” were reserved for findings supported by distinct participant sources. Findings appearing in multiple publications from the same parent cohort were described as within-cohort convergence and were counted only once when judging independent support for a domain-level conclusion.

### 2.9. Review Registration

The review was registered on PROSPERO (CRD420261458081).

## 3. Results

**Figure 2:**
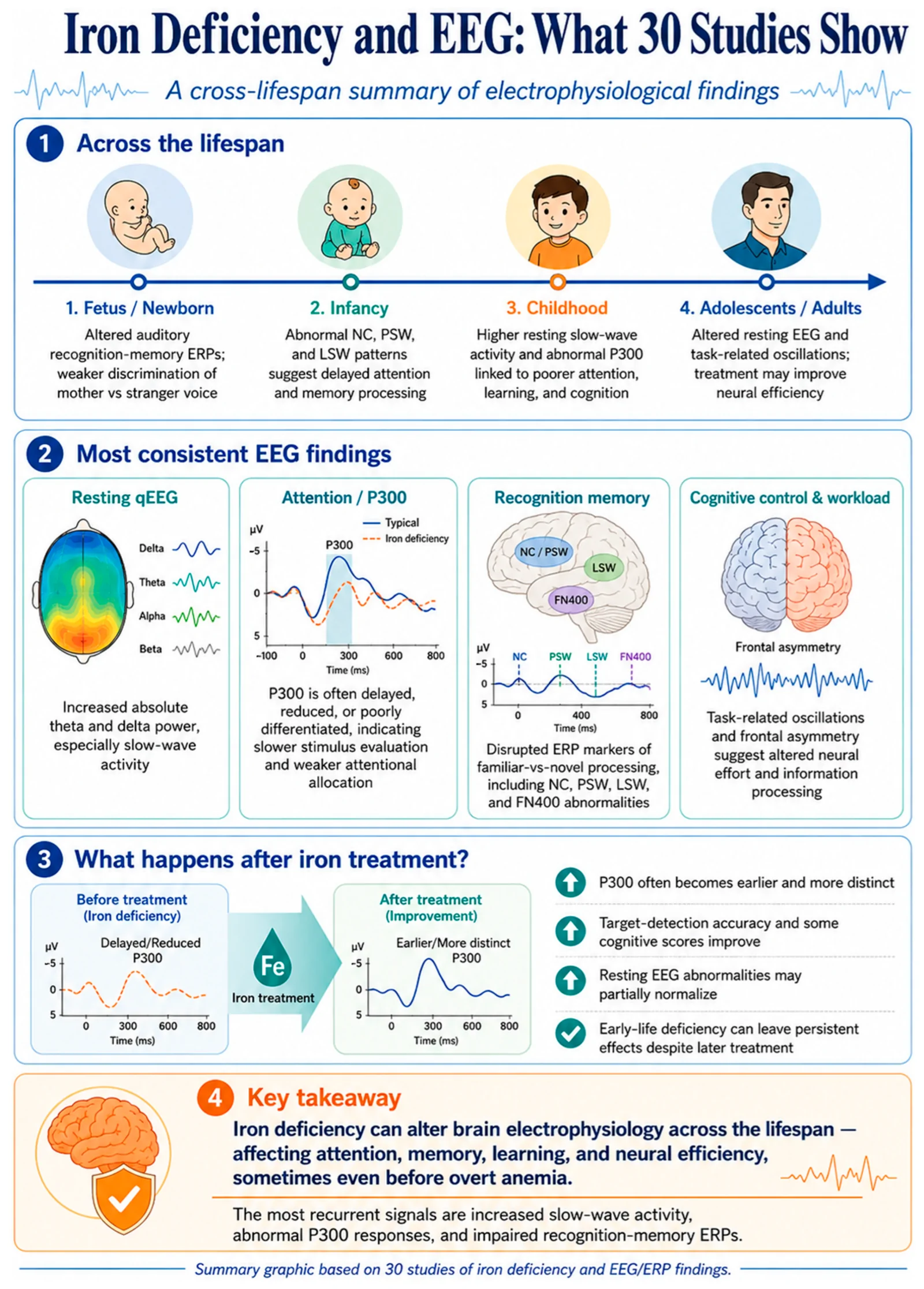
A cross-life span summary of electrophysiological findings.

Figure 1 presents the study-selection process in accordance with PRISMA 2020. The searches identified 101 records. Before screening, 52 duplicate records were removed, leaving 49 unique records for title and abstract screening. Eleven records were excluded at this stage because they clearly failed to meet the eligibility criteria. Full-text reports were therefore sought for 38 records; all 38 reports were successfully retrieved, and no reports were unavailable for assessment. The 38 full-text reports were assessed for eligibility. Eight reports were excluded: one because the participants did not have iron deficiency, two because they were review articles, two because they evaluated brainstem rather than cortical electrophysiological parameters, one because it was available only as a poster, and two because they were conference papers. Thirty reports were therefore retained in the complete narrative synthesis. Of these, 28 met the criteria for the core iron-specific evidence synthesis, whereas two were retained as contextual anemia evidence: one examined severe anemia associated with chronic kidney disease, and one examined mixed-etiology anemia in which iron-deficiency anemia accounted for 55.8% of cases but etiology-specific EEG results were unavailable. Thus, the complete synthesis comprised 30 reports, while conclusions specifically concerning iron deficiency were based primarily on the 28 core reports [31,32,33,34,35,36,37,38,39,40,41,42,43,44,45,46,47,48,49,50,51,52,53,54,55,56,57,58,59,60]. The characteristics and evidence classifications of all included reports are presented in Table 2. A cross-life span summary of electrophysiological findings is presented in Figure 2.

### 3.1. Participant Characteristics

The 30 reports included in the narrative synthesis represented heterogeneous populations across the human lifespan, ranging from newborn infants to adults. Twenty-eight reports constituted the core iron-specific evidence base, while two were retained as contextual anemia studies. The included populations varied substantially in sample size, developmental stage, sex distribution, and clinical characteristics. Detailed characteristics of individual studies are summarized in Table 2.

Three sets of included publications were identified as deriving from the same parent cohort or trial and therefore as wholly or partially non-independent. Studies [37,43] represented different ERP analyses from the same Chilean longitudinal cohort of children who had experienced iron-deficiency anemia during infancy. Studies [38,45,55] were derived from the same longitudinal birth cohort in Zhejiang Province, China, with EEG/ERP outcomes examined at different ages and using different paradigms. Studies [51,52] were both nested within the neurocognitive substudy of the same Benefits and Risks of Iron Supplementation in Children (BRISC) randomized trial in Bangladesh and analyzed different EEG outcomes with partially differing usable samples. These publications were retained because they contributed distinct developmental, paradigmatic, or outcome information, but they were not treated as independent replications. Consequently, the number of included reports should not be interpreted as an equivalent number of independent participant cohorts.

A substantial proportion of the evidence concerned fetal, neonatal, and early childhood iron status. Studies in newborns and young infants examined iron status using different biochemical indicators, including ferritin and zinc protoporphyrin-to-heme ratios, and included both infants with iron deficiency without overt anemia and those with iron-deficiency anemia [33,38,45,55]. Several longitudinal studies examined electrophysiological outcomes across infancy and later childhood, allowing developmental associations with early iron deficiency to be assessed [37,43,45].

School-age children and adolescents were also represented across observational and intervention studies. These populations included children with iron deficiency with or without anemia and participants enrolled in dietary or iron-supplementation interventions [31,32,36,39,42,44,49]. Some pediatric studies were conducted in socioeconomically disadvantaged settings, whereas others used individually matched iron-sufficient controls. The available evidence therefore encompassed both isolated depletion of iron stores and clinically apparent iron-deficiency anemia.

Adult studies were predominantly conducted in young women, although several investigations also included men and mixed-sex adult samples [35,40,41,46,48,50,53,59]. Adult populations included participants with iron deficiency without anemia as well as individuals with clinically defined iron-deficiency anemia. The two contextual anemia studies included adults with chronic kidney disease-related anemia or mixed-etiology anemia and were retained to provide information relevant to the potential contribution of anemia and reduced oxygen-carrying capacity rather than as evidence specific to iron deficiency [57,58].

The criteria used to define iron status varied considerably across studies. Investigators used serum iron, ferritin, zinc protoporphyrin-to-heme ratios, hemoglobin, or combinations of biochemical and hematological indicators, with thresholds differing according to age and study setting [31,32,33,38,44,45,55]. Consequently, the reviewed populations ranged from individuals with depleted iron stores and normal hemoglobin to participants with severe, clinically apparent iron-deficiency anemia. This heterogeneity is important when interpreting electrophysiological findings because effects associated with tissue iron deficiency may overlap with, but cannot necessarily be separated from, effects related to reduced oxygen-carrying capacity.

Most studies attempted to recruit otherwise healthy participants and excluded major neurological, sensory, systemic, or metabolic conditions that could influence electrophysiological measures. Nevertheless, some longitudinal pediatric cohorts retained differences in maternal, socioeconomic, or developmental characteristics, and infant studies frequently experienced attrition related to movement, crying, insufficient attention, or inadequate artifact-free recordings [33,37,38,43,45]. Overall, the evidence base was developmentally broad but demographically uneven, with substantial representation of infants, school-age children, and young women and comparatively limited evidence from older adults, adult men, and clinically diverse populations.

### 3.2. EEG Paradigms

The included studies used a broad range of electrophysiological paradigms, reflecting substantial differences in participant age, cognitive capacity, research objectives, and the hypothesized neurological consequences of iron deficiency. The methods could be divided into five principal categories: resting or spontaneous EEG, task-related spectral EEG, cognitive event-related potentials, infant recognition-memory and habituation paradigms, and visual evoked potentials. Some investigations combined more than one approach, most commonly resting quantitative EEG with an auditory P300 paradigm. The number of recording electrodes ranged from a single active site or a limited midline montage to high-density 64- or 128-channel systems. Consequently, the studies differed considerably in their capacity to characterize the spatial distribution of iron-related electrophysiological effects.

Resting-state and spontaneous EEG paradigms were used across different developmental stages to assess alterations in the organization, frequency composition, and maturation of ongoing cerebral activity [31,35,40,47,48,52,58]. In school-age children, quantitative EEG was used to examine absolute and relative power across conventional frequency bands, whereas studies in adults with iron-deficiency anemia combined resting quantitative EEG with conventional EEG assessment and, in some cases, repeated measurements following treatment [31,35,40]. Infant studies required developmentally adapted recordings, including spontaneous sleep EEG and resting recordings obtained while children passively viewed a neutral visual display [47,52]. These investigations examined conventional spectral measures as well as, in the most recent infant study, aperiodic and oscillatory components of the EEG spectrum [52]. Other studies focused on more specific aspects of cortical organization, including frontal alpha asymmetry in young women with iron deficiency without anemia [48] and qualitative abnormalities of background activity across different anemia etiologies and severities [58].

Taken together, these resting-state approaches differed considerably in recording conditions, developmental adaptations, montage, and analytical strategy. This heterogeneity limits direct comparison of individual spectral measures but provides complementary information about spontaneous cortical organization across age groups [31,35,47,48,52,58].

Task-related spectral EEG was used in two studies to examine whether iron status influenced cortical oscillatory activity during changing emotional or cognitive demands [45,46]. In infants, high-density EEG was recorded across neutral, positive-interaction, and socially challenging conditions, with analyses focusing on infant-specific frontal alpha asymmetry [45]. In adults, spectral EEG was assessed during progressively increasing cognitive workload, allowing changes in oscillatory activity to be considered alongside behavioral performance and metabolic demands [46]. These paradigms therefore extended the resting-state findings by examining whether electrophysiological differences associated with iron status were also evident during active emotional or cognitive engagement.

Cognitive ERPs constituted the largest group of paradigms and were used to examine several stages of information processing, including sensory encoding, attentional allocation, memory updating, response inhibition, and executive control. The most frequently used paradigm was the auditory oddball task, which was applied in children, adolescents, and adults with iron deficiency or anemia [35,36,40,42,50,57,59,60]. These studies primarily examined the P300 component, although earlier N100, P200, and N200 components were also assessed in some investigations [35,40].

Across the auditory oddball studies, P300 was generally recorded using relatively limited midline montages, most commonly involving Cz or Fz-Cz-Pz, making the paradigm suitable for assessing timing and the amplitude of higher-order stimulus processing but limiting detailed assessment of distributed or lateralized cortical effects [35,36,40,42,50,57,59,60]. The paradigms varied in stimulus characteristics, recording procedures, and treatment designs, including both observational comparisons and repeated assessments following iron or anemia treatment [35,40,57,60].

Working-memory processing was examined using a visual Sternberg-type memory-search task in school-age children [44]. The paradigm compared lower and higher memory loads and allowed ERP responses to be evaluated in relation to iron status and supplementation. This study therefore provided evidence concerning the electrophysiological correlates of memory maintenance and search rather than the more general attentional processing assessed by conventional oddball paradigms [44].

Several studies used paradigms that placed greater demands on working memory or executive control. Otero et al. [44] used a visual Sternberg-type memory-search task. Children memorized sets containing either three or five digits and then decided whether a subsequently presented probe digit had appeared in the set. The three-digit condition represented lower working-memory load, whereas the five-digit condition required greater maintenance, search, and decision-related processing. ERPs were calculated only from correct responses and compared across memory-load conditions, iron-status groups, and pre- versus post-supplementation assessments.

Raz, Koren, and Levin [41] recorded high-density EEG during a visual task-switching paradigm. A centrally presented digit was displayed in either black or blue. Depending on its color, participants judged whether it was greater or smaller than five or whether it was odd or even. Trials were classified as switch trials when the color and decision rule changed and as nonswitch trials when the same rule remained active. The paradigm was designed to assess flexible task-set updating, executive control, and the additional neural demand imposed by switching between rules.

Response inhibition was examined in a Chilean longitudinal study using a Go/No-Go paradigm [43]. The analysis focused on N2 and P300 responses during response inhibition, allowing early conflict detection and later allocation of processing resources to be assessed. Importantly, the study examined whether electrophysiological differences persisted in children who had experienced iron-deficiency anemia during infancy [43].

Broader cognitive batteries were used in dietary-intervention studies from Rwanda and India [39,49]. These paradigms combined measures of processing speed, attention, response inhibition, working memory, recognition, and executive control with continuous EEG recording. Analyses included early visual ERP components as well as oscillatory activity, allowing the studies to examine electrophysiological changes across multiple stages of information processing and their potential relationship with changes in iron status and cognitive performance [39,49].

Error monitoring and feedback processing were examined in a high-density EEG study using an intensive visual category-learning paradigm [53]. The study assessed error-related and feedback-related ERP components during rule-based and information-integration learning and additionally examined changes in neural efficiency across repeated practice. This paradigm provided evidence concerning electrophysiological processes involved in performance monitoring, feedback processing, and reinforcement-related learning [53].

Recognition-memory and habituation paradigms were particularly prominent in neonatal and infant studies [33,34,38,45,51]. These studies used auditory and visual stimuli to assess neural differentiation between familiar and novel information and included maternal-voice, face-recognition, and passive auditory roving-oddball paradigms. In newborns, auditory ERPs were used to examine responses to maternal versus unfamiliar voices, whereas infant face-recognition studies focused on the Nc and later slow-wave responses [33,34]. Related maternal-voice paradigms in young infants examined P2 and later slow-wave activity as indices of recognition-memory processing [38]. High-density face-recognition recordings at later stages of infancy similarly assessed frontal and temporal ERP responses to familiar and unfamiliar faces [45].

Recognition-memory paradigms were also applied beyond infancy. A school-age word-recognition study examined FN400 and P300 responses during old–new recognition, providing information about familiarity and memory updating [37]. In contrast, the Bangladeshi habituation study used a passive auditory roving-oddball paradigm to assess neural discrimination of novel sounds and attenuation of responses to repeated stimuli without requiring an overt behavioral response [51]. Thus, although these paradigms differed substantially in stimulus type and developmental applicability, they converged on the assessment of early sensory differentiation, recognition memory, and neural adaptation to repeated information.

Visual evoked potentials were examined in two studies involving infants and young children with iron-deficiency anemia [54,56]. Both used monocular visual stimulation and assessed stimulus-evoked waveform latencies as indicators of visual pathway conduction and maturation. Although the specific recording procedures differed between studies, the primary outcome was delayed VEP timing rather than alterations in spontaneous cortical activity or higher-order cognitive processing [54,56]. These findings therefore represent a distinct electrophysiological domain and are considered separately from EEG spectral and cognitive ERP measures.

### 3.3. EEG Spectral Power and Oscillatory Activity

Studies assessing resting-state and task-related EEG consistently indicated alterations in oscillatory activity associated with iron deficiency, although the direction and magnitude of effects varied according to age, anemia status, recording conditions, and analytical approach. The most recurrent pattern was increased slow-wave activity, particularly in the delta and theta ranges, accompanied in several developmental groups by reduced or altered alpha activity [31,47].

In school-age children with iron deficiency without overt anemia, increased absolute theta power was observed across widespread cortical regions, while absolute delta power was particularly elevated over frontal sites [31]. Similar increases in delta and theta activity, together with reduced alpha power, were reported in infants with iron-deficiency anemia [47]. In the latter study, four months of iron supplementation was followed by a reduction in theta activity and an increase in alpha power, with most treated infants subsequently falling within the laboratory-defined normal EEG range. These findings suggest that increased slow-wave activity may represent a relatively recurrent electrophysiological feature of iron deficiency during development, although the available evidence does not establish a uniform spectral signature.

Alterations in alpha organization were also reported, but findings were less consistent than those for slow-wave activity. Young women with low ferritin showed greater frontal alpha power at F3 than iron-sufficient controls, whereas infants with persistent iron deficiency demonstrated altered frontal alpha asymmetry, primarily related to reduced right-frontal alpha power [45,48]. During cognitively demanding visual tasks, iron-deficient women additionally showed greater alpha suppression and attenuated task-related theta and gamma responses as stimulus complexity increased [46]. These findings suggest that iron status may influence both baseline cortical organization and the dynamic recruitment of oscillatory activity during cognitive demands.

Treatment studies provided partially convergent but not uniform evidence for normalization of oscillatory activity. In adults with iron-deficiency anemia, iron treatment was followed by reductions in delta, theta, beta, and broadband power in several posterior and temporal regions, although the original report contained an internal inconsistency regarding the direction of some spectral changes [35]. This study was judged to be at critical risk of confounding because it used an uncontrolled pre–post design. Accordingly, the apparent spectral normalization was treated as low-confidence supportive evidence rather than proof of an iron-specific treatment effect, because temporal change, regression to the mean, correction of anemia, and other nonspecific factors could not be separated from the effect of iron replacement. In a randomized trial in young children, iron supplementation increased central mu-alpha power relative to placebo, whereas most other spectral measures were unaffected and the effect was no longer evident nine months after supplementation [52]. Thus, treatment-related changes in spectral activity appear possible but are not sufficiently consistent to support a specific electrophysiological marker of treatment response.

Overall, the available evidence supports a pattern of increased slow-wave activity and altered alpha organization, with greater consistency for delta/theta abnormalities than for alpha changes. However, differences in developmental stage, anemia severity, EEG methodology, and spectral definitions limit direct comparison across studies. The two contextual anemia studies were considered separately and were not used as independent evidence of an iron-specific spectral effect.

### 3.4. P300 Latency and Amplitude

P300 responses were examined across auditory and visual oddball paradigms, working-memory tasks, word-recognition procedures, and Go/No-Go paradigms. Across the available studies, prolonged P300 latency was the most recurrent finding, whereas changes in P300 amplitude were less consistent and appeared to depend on the severity of iron deficiency or anemia, task demands, and developmental stage [36,40,42,50,59,60].

Prolonged P300 latency was reported in children with iron-deficiency anemia, young women with iron-deficiency anemia, and adults with severe iron-deficiency anemia [36,40,50,59,60]. In several studies, longer latency was associated with lower hemoglobin or hematocrit, and in some cohorts with lower ferritin or serum iron concentrations [40,42,50]. However, the magnitude and statistical significance of group differences varied across studies. For example, one study of anemic girls did not identify a statistically significant between-group difference despite an inverse association between hematocrit and P300 latency [42]. These findings indicate that delayed P300 timing is relatively recurrent but is not sufficiently uniform to be regarded as a specific electrophysiological marker of iron deficiency. The contribution of study [59] to this pattern was specifically down-weighted because its classification of iron deficiency was judged to be at critical risk of bias. Participants were classified using hemoglobin-defined anemia without biochemical confirmation of iron deficiency; therefore, the marked prolongation of P300 latency observed in this study was considered evidence associated with anemia rather than independent evidence that iron deficiency itself prolongs P300 latency.

P300 amplitude showed greater heterogeneity. Amplitude was preserved in several studies involving children or young women with mild-to-moderate anemia [36,42,50], whereas reductions were observed in severe iron-deficiency anemia [40,59], in non-anemic iron-deficient children during visual target detection [32], and during more demanding working-memory conditions [44]. Smaller P300 amplitudes were reported in two analyses of the same Chilean longitudinal cohort assessed approximately 10 years after iron-deficiency anemia during infancy [37,43]. Because these reports examined overlapping participants using different cognitive paradigms, their agreement represents within-cohort convergence rather than two independent replications. The findings therefore support the possibility that some electrophysiological alterations persist beyond the period of acute deficiency, but independent longitudinal cohorts are required to establish the reproducibility and generalizability of this pattern. The reduced P300 amplitude reported in [59] was interpreted with the same restriction and was not counted as independent support for an iron-specific reduction in P300 amplitude.

Treatment-related changes in P300 parameters were also inconsistent. Iron supplementation was associated with shortening or normalization of P300 latency in some studies [35,60], whereas prolonged latency persisted after treatment in others [36,40]. However, the treatment-related P300 changes in [35] were assigned low interpretive weight because the critical risk of confounding in its uncontrolled pre–post design prevented separation of an iron-specific treatment effect from correction of anemia, repeated testing, and other time-dependent changes. Similarly, P300 amplitude increased following iron treatment in several studies [32,35,40], but remained largely unchanged in others [36,60]. Importantly, the randomized pediatric study demonstrated improvement in P300 latency in the iron-treated group, although some reduction was also observed in the placebo group, indicating that repeated testing or other nonspecific factors may have contributed to the observed change [60]. Therefore, treatment responsiveness should not be interpreted uniformly as evidence of a causal effect of iron replacement.

Longitudinal evidence further suggests that the timing of iron deficiency may be relevant. Children with a history of iron-deficiency anemia during infancy showed reduced P300 amplitudes during word-recognition and response-inhibition tasks at approximately 10 years of age [37,43]. These findings provide evidence of persistent developmental associations, but they should be interpreted cautiously because the observational design does not allow a direct causal attribution to earlier iron deficiency.

One additional study examined P300 responses in adults with chronic kidney disease-related anemia [57]. Because the anemia in this study was not attributable specifically to iron deficiency, these findings were considered contextual evidence regarding the possible contribution of anemia or impaired oxygen delivery, rather than evidence of an iron-specific electrophysiological effect.

Overall, the evidence indicates that P300 latency is more consistently altered than P300 amplitude in iron-deficient or anemic populations, with prolonged latency representing the most recurrent finding. However, substantial heterogeneity in diagnostic definitions, anemia severity, task paradigms, and treatment designs prevents establishment of P300 latency or amplitude as a specific biomarker of iron deficiency.

### 3.5. Early and Intermediate ERP Components: N1, P2, and N2

Early and intermediate ERP components showed heterogeneous associations with iron deficiency, with the clearest recurrent finding involving prolonged N2 latency. In contrast, N1 and P2 measures varied according to developmental stage, task characteristics, and treatment status [33,35,38,40,43,49,60].

N1 findings were inconsistent across studies. In adults with iron-deficiency anemia, N1 amplitude increased following iron treatment [35], while an iron-biofortified food intervention in adolescents was associated with changes in N1 amplitude across several attention and memory tasks [49]. In the placebo-controlled pediatric study, however, N1 latency shortened similarly in both the iron-treated and placebo groups, suggesting that the observed change was not specific to iron supplementation [60]. Neonatal studies also reported altered N1/P2-related responses under selected auditory conditions, but these findings varied according to the definition of iron deficiency and the type of stimulus used [33,38].

P2 findings were similarly heterogeneous. P2 amplitude increased following iron treatment in adults with iron-deficiency anemia [35,40], whereas no significant P2 differences were observed in newborns during simple auditory stimulation [33]. In infants with fetal–neonatal iron deficiency, altered P2 amplitude and hemispheric organization were reported during voice-processing conditions, although some associations were attenuated when alternative biochemical definitions of iron deficiency were applied [38]. These findings suggest that P2 alterations may be context-dependent rather than representing a uniform electrophysiological consequence of iron deficiency.

N2 latency showed a more consistent pattern. Prolonged N2 latency was observed in adults with severe iron-deficiency anemia and in children assessed at approximately 10 years of age after a history of iron-deficiency anemia during infancy [40,43]. In adults, N2 latency shortened following iron treatment in one study but remained prolonged in another [35,40]. In the pediatric placebo-controlled study, N2 latency and amplitude were not significantly affected by supplementation [60]. Thus, although delayed N2 processing appears in more than one developmental context, evidence for its reversibility with iron treatment remains inconsistent.

Overall, the available evidence suggests that N2 latency may be more consistently associated with iron deficiency than N1 or P2 measures, whereas N1 and P2 findings remain heterogeneous and strongly dependent on developmental stage and experimental context. The limited number of studies, differences in ERP paradigms, and inconsistent treatment responses prevent the identification of any early or intermediate ERP component as a specific biomarker of iron deficiency.

### 3.6. Recognition-Memory Components: NC, FN400, and Slow-Wave Activity

Recognition-memory paradigms provided evidence of altered electrophysiological differentiation between familiar and unfamiliar stimuli across several developmental populations. However, some apparently separate observations originated from the same longitudinal participant source. In particular, studies [38,45,55] represented different age-specific analyses from the Zhejiang longitudinal birth cohort and therefore do not constitute three independent replications. Across independent cohorts, the broader pattern involved absent, delayed, or developmentally atypical differentiation of familiar versus novel stimuli, although the direction and spatial distribution of effects varied according to age, timing of iron deficiency, and experimental paradigm [33,34,37,38,45,55]. In newborns and young infants, fetal or neonatal iron deficiency was associated with altered slow-wave responses during recognition of maternal versus unfamiliar voices. In studies using biochemical markers of fetal–neonatal iron status, iron-deficient infants showed reduced or absent mother–stranger differentiation in slow-wave activity compared with iron-sufficient infants [33,38]. Similar alterations were observed during face-recognition paradigms, although the pattern depended on age and on whether iron deficiency occurred during the fetal–neonatal period or later during infancy [34,45]. These findings suggest that iron status may influence the development of neural differentiation of socially relevant stimuli, but the heterogeneity of the reported spatial and temporal patterns limits identification of a single electrophysiological signature.

Longitudinal evidence further indicated that the timing of iron deficiency may be relevant. Infants with fetal–neonatal iron deficiency showed altered or absent recognition-related responses at selected developmental time points, whereas infants with postnatal iron deficiency sometimes retained partial differentiation between familiar and unfamiliar stimuli [45]. In the same longitudinal literature, some differences observed at nine months were no longer detectable at 18 months, indicating that at least part of the electrophysiological phenotype may change with maturation [45].

At school age, a history of iron-deficiency anemia during infancy was associated with abnormalities in the FN400 component during word recognition [37]. Children with previous infantile iron-deficiency anemia showed prolonged FN400 latency and reduced or absent differentiation between old and new words compared with controls. These findings suggest that some alterations in recognition-related neural processing may remain detectable years after the period of documented iron deficiency. However, because these observations derive from longitudinal observational data, they should be interpreted as persistent developmental associations rather than evidence of a direct causal effect of infantile iron deficiency.

Overall, recognition-memory studies suggest that iron deficiency, particularly during early development, may be associated with atypical neural differentiation of familiar and novel stimuli. The evidence is strongest for altered slow-wave and recognition-related ERP patterns in infants and for persistent FN400 abnormalities in children with a history of infantile iron-deficiency anemia. However, differences in age, timing of deficiency, biochemical definitions of iron status, and task paradigms substantially limit direct comparison across studies.

### 3.7. Error Monitoring and Feedback Processing: ERN, Pe, and FRN

Error monitoring and feedback processing were examined in a single study of 42 young adult women, including 22 participants with iron deficiency without anemia and 20 iron-sufficient controls [53]. Iron-deficient participants showed markedly reduced amplitudes of the error-related negativity (ERN), error positivity (Pe), and feedback-related negativity (FRN) during both rule-based and information-integration learning. ERN latency was additionally prolonged during rule-based learning, whereas no reliable latency difference was observed during information-integration learning [53].

The attenuation of ERN, Pe, and FRN amplitudes remained evident throughout extensive task practice, suggesting that the observed electrophysiological differences were not readily eliminated by repeated exposure to the task [53]. Higher ferritin concentrations were generally associated with larger error- and feedback-related ERP responses, supporting a continuous relationship between iron stores and these electrophysiological measures [53].

Because these findings are derived from a single observational study, they provide preliminary evidence that iron deficiency without anemia may be associated with altered error-monitoring and feedback-processing mechanisms. However, replication in independent cohorts and different age groups is required before these measures can be considered reliable electrophysiological indicators of iron deficiency.

### 3.8. Visual Evoked Potentials and Visual-Pathway Conduction

Visual evoked potentials (VEPs) were examined in two studies of infants and young children with iron-deficiency anemia [54,56]. Both studies reported delayed visual responses, with prolonged VEP latencies across early components of the visual evoked response [54]. In the comparative study, lower hemoglobin concentrations were strongly associated with longer VEP latencies, whereas relationships with serum iron and other iron-status parameters were less consistent [54].

In the treatment study, 12 weeks of oral iron supplementation were associated with a significant shortening of N2 latency, although post-treatment values remained slightly prolonged relative to the laboratory reference range [56]. This study was judged to be at critical risk of confounding because it lacked both an untreated iron-deficient comparison group and a healthy longitudinal control group. The observed latency shortening was therefore interpreted as suggestive of treatment responsiveness rather than evidence that iron replacement itself caused the improvement; developmental maturation, spontaneous temporal change, and correction of anemia could not be excluded as alternative explanations. Accordingly, study [56] contributed only limited weight to the conclusion that visual-pathway abnormalities may improve following treatment.

### 3.9. Auditory Novelty Detection and Habituation

Auditory novelty detection and habituation were examined in the neurocognitive substudy of the Benefits and Risks of Iron Supplementation in Children trial [51]. Children were randomized at approximately eight months of age to receive iron syrup, an iron-containing multiple micronutrient powder, or placebo for three months. EEG was recorded during a passive auditory roving-oddball paradigm at approximately 11 and 20 months of age.

Iron supplementation did not significantly affect the frontal or frontocentral deviant-minus-standard ERP response at either assessment point. Early auditory responses and the reduction in neural responsiveness during repeated stimulus presentation were likewise not significantly different between treatment groups [51]. The absence of an intervention effect was consistent across sensitivity analyses and subgroups defined by baseline iron deficiency, anemia, or iron-deficiency anemia [51].

Thus, in this randomized pediatric study, short-term iron supplementation did not produce measurable changes in auditory novelty detection or habituation, indicating that not all electrophysiological measures are responsive to correction of iron status during early childhood.

### 3.10. Visual P1 and Early Attentional Processing

Visual P1 responses were examined in three studies using attention, executive-control, and memory paradigms [39,41,49]. Findings were inconsistent. An observational study reported associations between erythrocyte-related indices and P1 amplitude, with lower MCH and MCV associated with larger posterior and more negative frontal P1 responses, particularly during more demanding task-switching conditions [41].

In contrast, two randomized dietary interventions found no significant effects of improved iron status on P1 amplitude or latency [39,49]. These findings indicate that although P1 measures may be associated with hematological indicators in observational analyses, current intervention evidence does not support a consistent effect of iron supplementation or iron-fortified foods on early visual attentional processing.

Overall, the evidence for P1 alterations is limited and heterogeneous, with observational associations not consistently supported by randomized intervention studies. P1 therefore cannot currently be considered a specific electrophysiological indicator of iron deficiency.

### 3.11. Conventional EEG Abnormalities and Background Organization

Conventional clinical EEG abnormalities were examined in two adult studies [40,58]. In adults with severe iron-deficiency anemia, abnormal EEG findings included diffuse or focal slowing, increased high-voltage slow-wave activity, reduced alpha activity, impaired background reactivity, and nonspecific paroxysmal or sharp-wave abnormalities [40]. Some of these abnormalities decreased following iron treatment, although not all electrophysiological abnormalities normalized.

A second study examined adults with anemia associated with chronic kidney disease and therefore did not provide iron-specific evidence [58]. In this mixed-etiology anemia sample, greater anemia severity was associated with more frequent diffuse slowing, reduced alpha activity, focal slowing, and paroxysmal discharges. These findings are relevant as contextual evidence for possible effects of anemia or impaired oxygen delivery, but they were not considered evidence of an electrophysiological effect specifically attributable to iron deficiency.

Overall, conventional EEG abnormalities were reported in adults with severe iron-deficiency anemia, but the evidence is limited to a small number of studies and includes heterogeneous EEG findings. The available data therefore do not support the use of conventional clinical EEG as a specific diagnostic marker of iron deficiency.

### 3.12. Risk of Bias Assessment

The risk of bias assessment of the included studies is presented in Table 3 (ROBINS-I) and Table 4 (RoB-2).

Risk-of-bias concerns were substantial across the non-randomized evidence base, particularly for confounding, participant selection, missing electrophysiological data, and, in selected studies, exposure classification and outcome reporting. Three studies were considered especially important for interpretation because at least one ROBINS-I domain was judged to be at critical risk of bias [35,56,59]. Studies [35,56] received critical judgments for confounding. Both used uncontrolled within-participant pre–post treatment designs, making it impossible to separate electrophysiological changes attributable specifically to iron treatment from temporal effects, regression to the mean, repeated assessment, developmental maturation where applicable, correction of anemia, or other concurrent clinical changes. Consequently, the post-treatment spectral and ERP changes reported in [35] and the shortening of visual-evoked-potential latency reported in [56] were retained as evidence of possible treatment responsiveness but were assigned low interpretive weight and were not regarded as independent evidence of a causal effect of iron replacement. Study [59] received a critical judgment for classification of the iron-deficiency exposure because participants were classified according to hemoglobin-defined anemia without biochemical confirmation of iron deficiency. Its finding of prolonged P300 latency and reduced P300 amplitude was therefore interpreted primarily as an association with anemia and was not counted as independent evidence of an iron-specific P300 abnormality. More generally, findings from studies with serious risk of bias were treated as supportive when they converged with better-controlled evidence but did not override null or conflicting findings from methodologically stronger studies.

The RoB 2 assessments similarly influenced interpretation of randomized evidence. High-risk domain judgments did not lead to study exclusion, but affected the weight assigned to the relevant outcomes. In particular, results subject to high risk of selective reporting or analysis-specific bias were treated as less definitive than directly randomized comparisons with fewer concerns. Thus, the narrative conclusions reported below reflect the consistency of findings after consideration of methodological quality rather than the number of nominally positive studies alone.

## 4. Discussion

### 4.1. Principal Findings and Integrative Interpretation

The reviewed evidence indicates that iron deficiency affects human brain electrophysiology at multiple levels, including spontaneous oscillatory activity, sensory conduction, attention, recognition memory, executive control, and performance monitoring. However, no single electrophysiological signature was consistently present across ages, deficiency severity, and recording paradigms. Findings varied with developmental stage, anemia status, the timing and duration of iron depletion, task demands, and the electrophysiological measure examined.

Three broad patterns emerged. First, iron deficiency during infancy and childhood was often associated with an immature or poorly organized resting EEG, including excessive delta and theta activity, reduced alpha activity, and background slowing. Second, delayed neural processing was reflected by prolonged P300, N2, FN400, and visual evoked-potential latencies. Third, iron-deficient participants frequently showed impaired adaptation of neural activity to task demands, including reduced theta and gamma recruitment with increasing cognitive complexity, weaker differentiation between target and non-target stimuli, atypical familiar–novel responses, and attenuated error- and feedback-related potentials.

These findings suggest that iron deficiency primarily disrupts neural efficiency and adaptive flexibility rather than simply increasing or decreasing EEG activity. Excessive resting theta may reflect delayed maturation or inefficient cortical organization, whereas task-related theta normally increases during working-memory maintenance and cognitive control. Iron deficiency may therefore produce elevated tonic slow activity while limiting the adaptive recruitment of theta during demanding tasks. Similarly, increased global activation during practice may coexist with smaller ERN, Pe, or FRN responses when neural activity is recruited diffusely rather than organized efficiently around specific processing events.

The relative preservation of some early sensory responses indicates that iron deficiency does not uniformly suppress neural responsiveness. Effects on P1, N1, and P2 latencies were inconsistent, particularly during simple stimulation, whereas abnormalities were more apparent during discrimination, recognition, working memory, inhibition, and feedback processing. The absence of effects on auditory novelty detection and habituation in the large supplementation trial is therefore important. It suggests that automatic sensory registration may be relatively resistant, while later processes requiring coordinated activity across distributed cortical and subcortical networks are more vulnerable.

### 4.2. Developmental Timing and Sensitive Periods

Developmental timing appears to be a major determinant of the electrophysiological phenotype. In infants, excessive slow-wave activity and reduced alpha power have been reported in association with early iron deficiency, while atypical NC and positive slow-wave responses during face recognition suggest altered progression through recognition-memory stages [33,38,45,52]. Persistent N2, P300, and FN400 abnormalities have been reported in two publications from the same Chilean longitudinal cohort of children who experienced iron-deficiency anemia during infancy [37,43]. Because the two reports examined overlapping participants using different paradigms—recognition memory and inhibitory control—they provide evidence of within-cohort convergence across cognitive domains rather than independent replication. These findings support an association between early-life iron deficiency and altered subsequent neural processing within this cohort, but confirmation in independent longitudinal samples is required before the persistence of these abnormalities can be considered robustly replicated. However, because the human studies were predominantly observational or longitudinal, these findings do not establish that early iron deficiency directly caused the later electrophysiological abnormalities.

Experimental evidence provides biological plausibility for this developmental interpretation. Perinatal iron deficiency has been associated with reduced cytochrome-c oxidase activity in metabolically active brain regions, including the hippocampus, cingulate cortex, and mediodorsal thalamus [61]. It has also been associated with alterations in the developmental expression of genes involved in energy metabolism, synaptic organization, and neuronal morphogenesis, with some changes persisting after iron repletion [62]. Experimental iron deficiency has further been associated with altered dendritic development in hippocampal CA1 pyramidal neurons and persistent changes in hippocampal DNA methylation and genes involved in neuronal development and intracellular signaling [63,64]. These findings provide potential biological explanations for the human electrophysiological associations, but the underlying cellular and molecular processes were not directly measured in the human EEG/ERP studies and should therefore be regarded as mechanistic hypotheses rather than demonstrated explanations of the observed electrophysiological findings.

These experimental observations are particularly relevant when considering recognition-memory abnormalities during infancy. The hippocampus and interconnected cortical regions undergo rapid maturation during late gestation and early infancy and are highly dependent on metabolic, synaptic, and structural processes. Disruption of these processes could plausibly contribute to altered recognition-related responses. However, the human EEG/ERP studies did not directly assess hippocampal structure, metabolism, dendritic morphology, or synaptic plasticity. Thus, altered familiar–novel differentiation, prolonged encoding, or atypical slow-wave responses should be interpreted as evidence of altered recognition-related neural processing rather than as direct evidence of hippocampal dysfunction.

Large-animal studies provide additional biological context. Postnatal dietary iron deficiency in piglets has been associated with alterations in corpus-callosum structure, white-matter microstructure, regional brain volumes, and hippocampal metabolite profiles [65]. Iron repletion improved some global measures but did not fully normalize regional volumetric or diffusion-tensor abnormalities [66]. Iron deficiency was also associated with reduced dendritic complexity in hippocampal pyramidal neurons. Experimental evidence concerning mitochondrial function, inhibitory neurotransmission, and glial metabolism provides plausible biological pathways through which iron deficiency could influence neural oscillations [61,62,67,68,69,70]. These findings suggest that structural and metabolic consequences of early iron deficiency may persist beyond hematological recovery. Nevertheless, animal findings cannot establish that the persistent electrophysiological abnormalities observed in humans are caused by incomplete structural recovery, altered connectivity, or persistent neuronal injury.

Long-term human studies provide complementary evidence. Children treated for iron deficiency during infancy have shown poorer cognitive, motor, educational, and socioemotional outcomes more than a decade later [13]. Follow-up to 19 years identified particularly adverse outcomes when infantile deficiency co-occurred with socioeconomic disadvantage [14], as well as persistent weaknesses in executive functioning and recognition memory [15]. Although these observational studies cannot fully exclude socioeconomic, nutritional, or caregiving confounding, their findings are consistent with the possibility that early-life iron deficiency is associated with long-term developmental differences. Recent neuroimaging studies have additionally reported altered patterns of neural recruitment and functional connectivity following early-life iron deficiency [71,72]. These findings provide complementary evidence of persistent neural differences, but they do not by themselves establish a specific cellular mechanism linking early iron deficiency to the electrophysiological abnormalities identified in this review.

Overall, the human evidence supports an association between early-life iron deficiency and persistent differences in electrophysiological and cognitive outcomes, particularly when deficiency occurs during periods of rapid brain development. Experimental and neuroimaging findings provide biologically plausible mechanisms involving metabolic function, neuronal development, white-matter organization, and network maturation. However, these mechanisms were not directly measured in the human EEG/ERP studies and therefore should be regarded as hypotheses requiring confirmation rather than established causal explanations.

### 4.3. Oscillatory Slowing, Cortical Organization, and Neural Efficiency

Increased resting delta and theta power in infants and children, together with diffuse slowing reported in severely anemic adults, suggests altered organization of cortical activity. During normal development, low-frequency dominance gradually decreases, while posterior alpha rhythms become more stable and differentiated. Persistent slow activity and reduced alpha organization may therefore be consistent with delayed maturation or reduced efficiency of neural network organization. However, these electrophysiological findings are not specific to iron deficiency and may also be influenced by developmental stage, anemia severity, vigilance, and other clinical factors.

Task-related findings provide additional evidence that the alteration may involve the ability to recruit neural resources under increased cognitive demand rather than generalized cortical slowing alone. In iron-deficient women, increases in theta and gamma activity were attenuated as task complexity increased. Because these oscillations are involved in cognitive control, working-memory coordination, and integration of task-relevant information, reduced task-related recruitment may indicate diminished oscillatory reserve. This interpretation remains functional rather than mechanistic, as the reviewed human studies did not directly measure the cellular processes underlying these oscillatory changes.

Experimental studies provide possible biological context for the observed electrophysiological pattern. Iron deficiency has been associated in animal models with alterations in mitochondrial energy metabolism and with changes in enzymes involved in inhibitory neurotransmission [18,61,62]. These findings provide biological plausibility for impaired neural synchronization and reduced capacity to support metabolically demanding activity. However, these cellular and molecular mechanisms were not directly measured in the human EEG studies included in this review. They should therefore be regarded as mechanistic hypotheses that may help interpret the electrophysiological findings, rather than as demonstrated explanations of the observed oscillatory abnormalities.

Similarly, alterations in frontal alpha asymmetry were observed in some non-anemic women and infants with recurrent deficiency. These findings should be interpreted cautiously because alpha lateralization is influenced by vigilance, affective state, task instructions, reference choice, and individual differences [73,74,75]. The recurring pattern may indicate altered frontal network organization, but replication in larger samples with standardized EEG methodology and concurrent behavioral measures is required.

Overall, the available evidence supports an association between iron deficiency and altered oscillatory organization, particularly increased slow-wave activity and reduced task-related recruitment of higher-frequency activity. The biological mechanisms responsible for these changes remain uncertain. Experimental evidence concerning mitochondrial function, inhibitory neurotransmission, and glial metabolism provides plausible pathways, but these mechanisms cannot currently be inferred directly from human EEG findings.

### 4.4. Neural Timing, Axonal Conduction, and Myelination

Latency prolongation was among the more consistent electrophysiological findings, affecting P300, N2, FN400, and visual evoked potentials. Although these components reflect different stages of sensory and cognitive processing, their prolonged timing may indicate less efficient temporal coordination of neural processing. However, the human studies included in this review did not directly measure axonal conduction, white-matter integrity, or myelination. Consequently, the electrophysiological findings should not be interpreted as direct evidence of impaired myelination.

Experimental evidence provides a plausible biological context for this interpretation. Iron is required for oligodendrocyte development, lipid metabolism, and myelin formation. Experimental iron deficiency has been associated with reductions in myelin-related proteins, alterations in brain phospholipid composition, and impaired oligodendrocyte maturation and myelination [76,77,78,79]. Animal studies have also reported alterations in white-matter development and diffusion-related measures following early iron deficiency, with some abnormalities persisting after dietary repletion [65,66]. These findings provide biological plausibility for a possible contribution of altered white-matter development to prolonged electrophysiological latencies, particularly when iron deficiency occurs during periods of rapid neural maturation. However, these structural and cellular changes were not directly assessed in the human EEG/ERP studies included in this review and therefore cannot be considered demonstrated mechanisms underlying the observed electrophysiological abnormalities.

Nevertheless, this interpretation remains indirect. Visual evoked-potential latency may provide relatively direct information about the timing of sensory-pathway transmission, whereas P300, N2, and FN400 latencies reflect the cumulative influence of sensory processing, attention, stimulus evaluation, memory access, and response selection. Therefore, similar latency prolongation across different ERP components does not necessarily imply a common alteration in axonal conduction or myelination.

The persistence of some latency abnormalities after hematological improvement may be compatible with slower recovery of neural systems than of circulating iron or hemoglobin. Experimental findings indicate that structural and developmental consequences of early iron deficiency may persist after nutritional repletion [65,66]. However, persistent ERP abnormalities in humans cannot by themselves establish incomplete myelin repair as the underlying mechanism, because developmental maturation, residual confounding, and differences in task performance may also contribute.

Overall, altered myelination and white-matter development represent plausible mechanisms that may contribute to delayed electrophysiological timing, particularly during early development, but this hypothesis requires direct testing with multimodal approaches combining EEG/ERP with structural or diffusion-based neuroimaging and longitudinal assessment.

### 4.5. Attentional Allocation and Task-Dependent Vulnerability

P300 latency was more consistently altered than amplitude across the reviewed studies, particularly in participants with iron-deficiency anemia. Prolonged latency may reflect slower stimulus evaluation, but P300 is a late and composite response influenced by attention, working memory, stimulus evaluation, and response selection. Therefore, prolonged P300 latency cannot be attributed specifically to reduced iron availability or impaired oxygen delivery on the basis of the available studies alone.

The dependence of some electrophysiological differences on task difficulty is nevertheless noteworthy. Group differences were often more apparent when memory load, stimulus complexity, inhibitory demands, or sustained cognitive effort increased. In iron-deficient participants, attenuated task-related theta and gamma responses were also reported during more demanding conditions. These findings are consistent with the possibility that iron deficiency may be associated with reduced efficiency of neural recruitment under higher cognitive demands. However, this interpretation remains functional and inferential, because the reviewed human studies did not directly measure neural resource allocation, compensatory capacity, or the cellular mechanisms underlying these oscillatory changes.

Intervention studies provide partially supportive but heterogeneous evidence. In young women, anemia severity was more strongly related to processing speed, whereas measures of iron deficiency were associated with cognitive accuracy, and cognitive outcomes improved following treatment [18]. A randomized trial in non-anemic iron-deficient adolescent girls also reported improvements in verbal learning and memory following supplementation [17]. These findings suggest that cognitive effects may arise through partially distinct pathways related to anemia and iron deficiency. However, cognitive improvement following treatment does not by itself establish a specific electrophysiological mechanism.

Early ERP findings provide some support for a greater vulnerability of later, cognitively demanding processing stages. N1 and P2 latency were often relatively preserved during simple auditory stimulation, whereas P2 amplitude, N2 latency, and P300 measures were more frequently altered when stimuli required selection, classification, memory, or response control. In contrast, P1 findings were inconsistent. This pattern is compatible with greater involvement of higher-order processing stages, although differences in task design and developmental stage limit direct comparison across studies.

Overall, the available evidence suggests that electrophysiological alterations may become more apparent as cognitive demands increase. However, the distinction between anemia-related slowing, iron-specific effects, and compensatory neural responses remains unresolved. Future studies should therefore assess iron status and hemoglobin simultaneously and combine electrophysiological measures with standardized behavioral indices of attention and executive functioning.

### 4.6. Recognition Memory and Hippocampal–Cortical Networks

Recognition-memory studies provided evidence of altered electrophysiological differentiation between familiar and unfamiliar stimuli during early development. Iron-sufficient newborns and infants generally showed distinct slow-wave responses to familiar and unfamiliar voices, whereas infants with suspected fetal or neonatal iron deficiency often showed reduced or absent differentiation. Later in infancy, iron-deficiency anemia was associated with altered developmental patterns of attention- and memory-related responses to familiar and unfamiliar faces. At school age, children with a history of infantile iron-deficiency anemia showed delayed FN400 responses and weaker old–new differentiation despite relatively preserved behavioral recognition accuracy. These findings indicate that early iron deficiency may be associated with persistent alterations in recognition-related neural processing, although they do not establish a specific underlying neural mechanism.

The hippocampus represents one plausible biological substrate for these findings, but this interpretation is based primarily on experimental evidence rather than direct measurement in the reviewed human electrophysiological studies. Experimental studies have reported alterations in CA1 dendritic development, hippocampal metabolic pathways, neuronal morphology, and developmental gene regulation following early-life iron deficiency [62,63,64]. Studies in piglets have additionally reported altered hippocampal metabolites [80], while experimental studies have demonstrated altered dendritic development in hippocampal CA1 pyramidal neurons [63]. These observations provide biological plausibility for a contribution of hippocampal structural and metabolic alterations to recognition-memory abnormalities. However, hippocampal structure and function were not directly measured in the human EEG/ERP studies included in this review, and the observed electrophysiological abnormalities therefore cannot be attributed specifically to hippocampal dysfunction.

Experimental evidence also suggests that iron availability can influence hippocampal synaptic function. Perinatal nutritional iron deficiency has been associated with altered basal hippocampal transmission, while iron chelation can affect NMDA-receptor-associated calcium signaling, ERK1/2 activation, and long-term potentiation [80,81]. Such findings indicate potential effects of iron status on synaptic plasticity and neuronal responsiveness. Nevertheless, differences between experimental models, developmental stages, and stimulation paradigms prevent these observations from being interpreted as a demonstrated explanation for the human recognition-memory ERP findings.

The absence or attenuation of familiar–novel differentiation may therefore reflect altered efficiency of recognition-related neural processing, but several mechanisms could contribute, including differences in sensory encoding, attention, memory formation, synaptic plasticity, and network maturation. Similarly, persistent FN400 and P300 abnormalities at school age may reflect altered developmental trajectories or compensatory processing, but the available longitudinal human studies cannot determine which neural mechanisms are responsible.

Overall, the recognition-memory findings provide some of the strongest evidence for developmental electrophysiological associations with early iron deficiency, particularly when altered familiar–novel differentiation is observed across multiple developmental stages. However, the proposed involvement of hippocampal morphology, synaptic plasticity, or hippocampal–cortical connectivity remains biologically plausible rather than directly demonstrated in the human electrophysiological literature [82]. Multimodal longitudinal studies combining EEG/ERP with structural and functional neuroimaging would be required to test these mechanisms directly.

### 4.7. Error Monitoring, Feedback Processing, and Dopaminergic Mechanisms

A single study in young women with non-anemic iron deficiency found attenuated ERN, Pe, and FRN responses, indicating altered electrophysiological processing of errors and external feedback despite normal hemoglobin [53]. The persistence of these differences during task practice, even as behavioral performance differences became less apparent, suggests that electrophysiological measures may detect alterations that are not necessarily reflected in overt task accuracy. However, replication in independent samples is required before these findings can be considered a reproducible feature of non-anemic iron deficiency.

Dopaminergic signaling represents one possible biological explanation for altered error- and feedback-related responses. Experimental studies have reported changes in dopamine receptor expression and regulation following iron deficiency, including alterations involving D1- and D2-related systems [83,84,85]. Because ERN and FRN generation involves interactions between reinforcement-related signals, basal-ganglia circuits, and medial frontal regions, altered dopaminergic signaling may provide one plausible biological pathway through which iron status could influence these electrophysiological responses. However, this interpretation is based on experimental evidence rather than direct measurement of dopaminergic function in the human study included in this review.

However, this interpretation remains mechanistic and inferential. The human study included in this review did not directly measure dopamine synthesis, receptor availability, dopamine transporters, or striatal function. Consequently, the observed attenuation of ERN, Pe, and FRN cannot be attributed specifically to dopaminergic dysfunction. Other processes, including altered synaptic transmission, excitation–inhibition balance, metabolic function, or neural synchronization, could also contribute to the observed electrophysiological differences [86,87].

The available evidence therefore supports an association between non-anemic iron deficiency and altered error- and feedback-related electrophysiological responses, but does not establish a dopaminergic mechanism in humans. Larger studies combining EEG/ERP with direct or indirect measures of dopaminergic function and longitudinal assessment of iron status are needed to determine whether dopaminergic alterations contribute to these findings.

### 4.8. Iron-Specific Neural Effects and Anemia-Related Oxygen Limitation

An important consideration in interpreting the reviewed electrophysiological findings is the distinction between the effects of tissue iron deficiency and those associated with anemia or reduced oxygen-carrying capacity. The two processes frequently coexist, but they are not physiologically equivalent. Iron is required for mitochondrial metabolism, neurotransmitter synthesis, myelin formation, and other cellular processes independently of its role in hemoglobin synthesis. Conversely, anemia may affect neural function through reduced oxygen delivery even when tissue iron deficiency is not the primary mechanism [88].

Several findings in the reviewed literature provide evidence that electrophysiological alterations may occur in the absence of overt anemia. Changes in spectral EEG activity, frontal alpha organization, and error- and feedback-related ERPs were reported in participants with iron deficiency but normal hemoglobin concentrations [31,45,48,53]. These findings are particularly relevant because they suggest that some electrophysiological associations cannot be explained solely by reduced oxygen-carrying capacity. However, the number of studies specifically examining non-anemic iron deficiency remains limited, and many studies did not simultaneously assess all relevant markers of iron status and hematological function.

Other electrophysiological abnormalities were observed primarily in participants with iron-deficiency anemia. Prolonged P300, N2, and VEP latencies and diffuse EEG slowing were reported in anemic populations [35,36,40,54,56,59,60]. In some studies, the magnitude of electrophysiological alterations was more closely related to hemoglobin or haematocrit than to ferritin or serum iron [40,54]. These findings raise the possibility that impaired oxygen delivery contributes substantially to some latency and background-EEG abnormalities. Accordingly, such findings should not be interpreted as evidence of an iron-specific electrophysiological effect unless iron status has been independently assessed and its association with the outcome demonstrated.

The distinction is also relevant when interpreting treatment studies. Improvements in electrophysiological parameters following iron supplementation may reflect restoration of tissue iron availability, correction of anemia, or both. Where treatment studies lacked placebo controls or included participants with concurrent anemia, the relative contribution of these mechanisms cannot be established. The observation that some electrophysiological abnormalities were present in non-anemic iron-deficient participants provides support for an iron-specific contribution, but it does not exclude additional effects related to subtle hematological changes or other clinical factors.

The contextual studies involving anemia of non-iron-specific or mixed etiology provide complementary evidence but should remain analytically separate from the core iron-deficiency evidence. In particular, the study of chronic kidney disease-related anemia [58] may illustrate the potential electrophysiological consequences of anemia and altered oxygen delivery, but it cannot be used to infer a specific effect of iron deficiency. Similarly, other studies involving mixed or incompletely characterized anemia should be interpreted as contextual rather than iron-specific evidence.

Overall, the available evidence suggests that both iron-dependent mechanisms and anemia-related reductions in oxygen delivery may contribute to electrophysiological abnormalities, but their relative contributions cannot currently be determined with confidence. Future studies should therefore characterize ferritin and other indicators of iron status together with hemoglobin, haematocrit, transferrin saturation, and relevant inflammatory markers, and should analyze electrophysiological outcomes separately according to iron deficiency with and without anemia.

### 4.9. Treatment Responsiveness and Incomplete Reversibility

Several studies reported changes in electrophysiological measures following iron supplementation, including reductions in slow-wave activity, shortening of ERP or VEP latencies, and increases in selected ERP amplitudes [32,35,40,52,56,60]. These findings suggest that at least some electrophysiological alterations associated with iron deficiency or iron-deficiency anemia may be modifiable following improvement in iron status. However, the strength of this evidence varies substantially according to study design.

The strongest evidence comes from randomized or placebo-controlled studies. In the randomized pediatric study, iron supplementation was associated with changes in selected P300 and mu-alpha measures, although some electrophysiological changes were also observed in the placebo group [52,60]. This pattern indicates that repeated testing, developmental maturation, or other nonspecific effects may have contributed to the observed changes. Similarly, not all electrophysiological outcomes were affected by supplementation, and some abnormalities persisted despite treatment.

Several uncontrolled pre–post studies reported normalization or partial improvement in EEG or ERP measures after iron treatment [32,35,40,56]. These findings are potentially important but cannot by themselves establish causality because maturation, regression to the mean, repeated assessment, changes in clinical status, and other time-dependent factors cannot always be excluded. In addition, some treatment studies included participants with iron-deficiency anemia, making it difficult to determine whether electrophysiological improvement reflected restoration of tissue iron availability, correction of anemia, or both.

The persistence of electrophysiological differences after treatment in some developmental cohorts further complicates interpretation. Persistent abnormalities may reflect incomplete recovery, developmental timing, or long-term associations established during sensitive periods of brain development. However, the available longitudinal human evidence does not allow these possibilities to be distinguished reliably.

Treatment responsiveness should therefore be interpreted as supportive but not definitive evidence of a relationship between iron status and electrophysiological function. A causal interpretation would require adequately powered randomized trials with placebo or appropriate control groups, standardized EEG/ERP protocols, repeated assessments, and simultaneous measurement of iron status and hematological parameters.

Overall, the available intervention evidence suggests that some electrophysiological measures may change following correction of iron deficiency, but no EEG or ERP measure has demonstrated sufficiently consistent and specific treatment responsiveness to serve as a validated biomarker of iron repletion or neural recovery.

## 5. Limitations and Future Directions

Although iron deficiency is associated with alterations in resting EEG, neural oscillations, ERPs, attention, memory, learning, and executive control, the literature remains highly heterogeneous. Differences in age, developmental stage, deficiency severity and duration, anemia status, cognitive paradigms, EEG methodology, and treatment design prevent identification of a single electrophysiological signature. Current findings should therefore be interpreted as converging evidence of disrupted neural function rather than as a validated diagnostic or prognostic biomarker.

Risk of bias further limits the strength of causal interpretation. Serious ROBINS-I concerns were frequent across the observational literature, particularly because iron status was not randomly assigned and could be associated with socioeconomic, nutritional, developmental, inflammatory, and hematological factors that also influence brain function. Importantly, three studies contained critical risk-of-bias judgments that materially reduced their contribution to the synthesis [35,56,59]. The uncontrolled pre–post studies [35,56] could demonstrate temporal co-occurrence between iron treatment and electrophysiological improvement but could not establish that treatment caused the observed changes. Study [59], in contrast, could demonstrate an association between anemia and P300 abnormalities but could not establish that the association was attributable specifically to iron deficiency because iron status was not biochemically confirmed. These studies were therefore retained for completeness but were explicitly down-weighted and were not used as stand-alone support for causal or iron-specific conclusions. The principal conclusions of this review consequently rely on patterns that remained evident after greater weight was assigned to biochemically characterized, controlled, longitudinal, or randomized evidence.

An additional limitation concerns non-independence among several publications derived from the same longitudinal cohorts or randomized trial. Specifically, studies [37,43] originated from the same Chilean longitudinal infant-IDA cohort, studies [38,45,55] from the same Zhejiang longitudinal birth cohort, and studies [51,52] from the same BRISC neurocognitive trial population. Although these reports contributed distinct developmental time points, electrophysiological paradigms, outcomes, or analytic subsets, they do not represent independent replications. Counting publications rather than parent cohorts could therefore exaggerate the apparent recurrence of selected recognition-memory, P300, developmental, or treatment-related findings. In the present synthesis, agreement among overlapping publications was treated as within-cohort convergence, whereas claims of replication or cross-study consistency were based on independent participant sources. Future studies should clearly report parent-cohort provenance and prioritize replication in independent populations.

A major limitation is the absence of a standardized definition of iron deficiency. Studies used different thresholds and combinations of ferritin, serum iron, transferrin saturation, soluble transferrin receptor, zinc protoporphyrin-to-heme ratio, hemoglobin, haematocrit, and red-cell indices. Some examined non-anemic deficiency, whereas others included iron-deficiency anemia or selected participants primarily on the basis of anemia. This distinction is important because electrophysiological abnormalities may arise from tissue iron depletion, impaired mitochondrial and neurotransmitter function, reduced myelination, or anemia-related cerebral oxygen limitation. When iron stores and hemoglobin improved simultaneously, their separate contributions could rarely be determined. Future studies should distinguish non-anemic iron deficiency from iron-deficiency anemia and analyze iron biomarkers continuously rather than only categorically.

Iron markers are also influenced by inflammation, diet, time of day, age, sex, pregnancy, smoking, and altitude. Ferritin may overestimate stores during inflammation, while serum iron varies considerably. Future studies should use standardized panels including hemoglobin, ferritin, transferrin saturation, soluble transferrin receptor, mean corpuscular volume, and, where appropriate, zinc protoporphyrin-to-heme ratio. Ferritin should be interpreted alongside *C-*reactive protein and α1-acid glycoprotein. Diet, menstrual blood loss, pregnancy, infection, parasitic disease, lead exposure, altitude, and supplementation should also be recorded. Hepcidin and the soluble transferrin receptor-to-ferritin index may further improve characterization of biologically relevant iron restriction.

Small samples were common, particularly in infant and child ERP studies, with some groups containing fewer than 20 participants and longitudinal analyses fewer than ten. Small samples reduce power, destabilize effect estimates, and limit analysis of sex, developmental timing, severity, socioeconomic background, and nutritional comorbidity. Attrition was also substantial because of movement, crying, insufficient trials, artifacts, low task accuracy, and incomplete follow-up. Excluding participants with poor performance or unusable EEG may preferentially remove those with the greatest impairment. Future studies should use prospective power calculations, larger multicentre samples, transparent reporting of data loss, and appropriate missing-data methods.

Most evidence is observational, cross-sectional, or derived from non-randomized pre–post studies. These designs cannot fully separate iron deficiency from socioeconomic disadvantage, maternal education or depression, food insecurity, malnutrition, inflammation, infection, toxicant exposure, pregnancy complications, and differences in caregiving or educational opportunity. Prenatal studies face additional confounding by maternal diabetes, hypoxia, prematurity, and altered fetal growth. Large prospective cohorts beginning in pregnancy and repeatedly assessing iron status, development, behavior, and electrophysiology are needed to determine how the timing, duration, and recurrence of deficiency shape neural outcomes.

Treatment findings also require caution. Some studies reported improvement in P300, memory-related slow waves, or resting EEG after supplementation, but many lacked placebo-treated deficient groups, repeated healthy-control testing, or iron-only interventions. Improvement may therefore partly reflect maturation, practice, regression to the mean, or better general health. Persistent abnormalities may represent either insufficient biochemical recovery or lasting developmental effects. Conversely, null findings in large trials may reflect inclusion of children without deficiency, exclusion of severe anemia, short treatment duration, or insensitive EEG measures.

Future trials should recruit participants with confirmed deficiency, stratify by anemia severity, and prespecify subgroup analyses. Where ethical, they should use masked placebo or active comparators, objectively assess adherence, document diet and additional supplementation, and repeatedly measure ferritin and hemoglobin. Serial EEG assessments could clarify whether biochemical, electrophysiological, and behavioral recovery occur at different rates. Long-term follow-up is essential to determine whether persistent abnormalities reflect permanent change, delayed maturation, or slow recovery.

EEG acquisition and analysis were also highly inconsistent. Studies differed in paradigms, electrode density, reference montage, filters, sampling rate, epoch duration, artifact handling, trial thresholds, frequency-band definitions, baseline correction, electrode selection, and statistics. Resting-state studies reported absolute or relative power, predefined bands, peak measures, or periodic activity after removal of the aperiodic component. These differences may explain apparently conflicting results, while some older studies reported insufficient preprocessing details or inconsistencies between numerical findings and interpretation.

The field would benefit from harmonized, age-appropriate protocols. Resting EEG should include standardized eyes-open and eyes-closed conditions, adequate duration, vigilance monitoring, developmental frequency bands, and separate analysis of periodic and aperiodic activity. ERP research should use a core set of paradigms examining attention, P300 generation, recognition memory, working memory, inhibition, and error or feedback processing. Acquisition, referencing, artifact handling, trial retention, component windows, and regions of interest should be defined prospectively and reported fully. High-density EEG, source reconstruction, time–frequency analysis, connectivity measures, and multivariate methods may reveal network-level effects, but exploratory findings should be separated from confirmatory analyses and independently validated.

Many studies tested numerous electrodes, components, time windows, bands, and behavioral outcomes without sufficiently strict correction for multiple comparisons. Combined with small samples and flexible analyses, this increases the risk of false-positive and selectively reported results. Future studies should preregister hypotheses and analysis plans, report effect sizes and confidence intervals, publish null findings, and share de-identified EEG data, code, task materials, and detailed iron measures. Replication of increased slow-wave power, P300 abnormalities, atypical recognition-memory responses, and reduced error-related activity should precede their interpretation as stable mechanistic markers.

Finally, EEG outcomes should be integrated more closely with behavior and clinical function. Some studies inferred cognitive impairment from ERPs without demonstrating associations with task performance, neuropsychological scores, academic functioning, or symptoms. In other cases, normal performance coexisted with persistent neural abnormalities, suggesting compensation or subclinical inefficiency. Future studies should test whether EEG changes mediate cognitive recovery and predict treatment response. Combining EEG with MRI, myelin-sensitive imaging, cerebral blood-flow measures, and markers of dopaminergic, mitochondrial, and inflammatory function may help distinguish the mechanisms through which iron deficiency affects the brain.

## 6. Conclusions

The available evidence indicates that iron deficiency is associated with alterations in electrophysiological measures of brain function, but no specific EEG or ERP biomarker of iron deficiency can currently be established. After accounting for overlapping cohorts, the most recurrent findings across independent participant sources include increased delta/theta activity, alterations in alpha organization, prolonged P300 and N2 latencies, and abnormalities in recognition-related ERP responses, although the degree of independent replication differs substantially across these domains. However, the strength and consistency of these findings vary considerably according to developmental stage, severity and duration of iron deficiency, presence of anemia, EEG/ERP paradigm, and study quality.

The current evidence does not support routine EEG or ERP testing as a screening or diagnostic investigation for iron deficiency. Iron deficiency should continue to be identified and monitored using established hematological and biochemical measures rather than electrophysiological testing. Electrophysiological assessment may nevertheless have value in selected clinical or research settings, particularly when the aim is to investigate possible functional consequences of prolonged or severe deficiency rather than to establish the diagnosis of iron deficiency itself.

From the available evidence, the most promising electrophysiological approaches are spectral EEG analysis and P300-based paradigms, with recognition-memory ERPs and VEPs providing additional information in selected developmental populations. Evidence concerning error-monitoring ERPs, P1, and other individual components is currently too limited or inconsistent to support their use as specific indicators of iron deficiency. During infancy and early childhood, electrophysiological measures may be particularly informative for studying developmental consequences of prolonged or early-life iron deficiency, whereas evidence in older children and adults is less consistent and does not justify routine clinical application.

Treatment-related changes in EEG and ERP measures suggest that some electrophysiological alterations may be modifiable following improvement in iron status. However, these findings do not establish that electrophysiological abnormalities are caused exclusively by iron deficiency, particularly when anemia is present or when studies lack placebo or untreated control groups. Persistent developmental associations reported after early-life deficiency further support the need for longitudinal research but should not be interpreted as proof of irreversible neural injury.

Overall, electrophysiological methods should currently be regarded as complementary research and potentially supportive clinical tools rather than diagnostic tests for iron deficiency. Future studies should use standardized EEG/ERP protocols, distinguish iron deficiency with and without anemia, account for overlapping cohorts, and combine electrophysiological measures with detailed biochemical, behavioral, and neurodevelopmental assessments. Well-designed longitudinal and randomized studies are needed to determine whether specific electrophysiological measures can ultimately provide clinically useful information about the functional consequences or recovery of iron deficiency.

## Figures and Tables

**Table 1:** Main causes of iron deficiency across the lifespan.

Main Cause	Main Mechanism	Examples/Populations
Inadequate dietary intake	Insufficient intake or low bioavailability of dietary iron	Infants, children, adolescents, restrictive diets
Increased physiological requirements	Increased iron requirements during periods of rapid growth or pregnancy	Infancy, childhood, adolescence, pregnancy
Chronic blood loss	Persistent iron loss exceeding replacement	Menstrual blood loss, gastrointestinal bleeding
Impaired intestinal absorption	Reduced intestinal uptake of dietary iron	Gastrointestinal disorders, malabsorption
Inflammation/functional iron deficiency	Hepcidin-mediated iron sequestration and impaired mobilization	Chronic inflammatory conditions
Increased developmental demand	High iron requirements during rapid tissue and neural development	Fetal life, infancy, early childhood

**Table 2:** Studies included in review.

Study	Life Stage/Age	Sample	Design	Iron Status/ Exposure	EEG Paradigm	EEG/ERP Measures	Main Electrophysiological Findings	Treatment/ Longitudinal Response	Behavioral/ Cognitive Findings	Interpretive Notes/Limitations	Primary Modality	Modality Family	Intervention
[31]	Children (6–12 years)	33 iron-deficient, non-anemic children and 33 age-, sex-, and socioculturally matched iron-replete controls.	Matched cross-sectional study.	Serum iron <60 µg/dL; lower ferritin and hemoglobin, but no overt anemia.	10 min eyes-closed resting EEG; 19 electrodes; linked-ear and surface-Laplacian montages; FFT of artifact-free epochs.	Absolute and relative delta, theta, alpha, and beta power.	Iron deficiency was associated with globally increased absolute theta power in the Laplacian montage and increased frontal absolute delta power in the referential montage. Relative power did not differ.	Not assessed.	Lower WISC-R verbal, performance, and full-scale IQ; markedly poorer dynamic learning performance.	Suggests diffuse cortical slowing, with a particularly frontal delta abnormality; no sex interaction.	Resting qEEG	Resting/conventional EEG	No
[32]	Children (8–10 years)	10 iron-deficient children completing the full protocol and 10 matched iron-replete controls.	Matched longitudinal supplementation study; controls tested once.	Serum iron <60 µg/dL; generally non-anemic depleted iron stores; 5 mg/kg/day elemental iron for up to 3 months.	Visual continuous-performance oddball task with animal images; target crab; linked-ear referenced EEG.	Visual P300 amplitude and latency; target–nontarget ERP differentiation.	Untreated iron-deficient children showed an almost absent centroparietal P300 and minimal target–nontarget differentiation; P300 latency was not meaningfully altered.	Supplementation produced a clear P300 and substantial normalization, although a residual reduction remained at Pz.	Fewer correct target responses before treatment; accuracy improved to near-control levels after supplementation; reaction time unchanged.	Pattern points to reduced attentional allocation/stimulus discrimination rather than slowed stimulus evaluation.	Visual ERP	ERP	Yes
[33]	Newborns (~15 days)	32 infants of diabetic mothers; 23 with usable ERP data (9 suspected brain iron deficient, 14 brain iron sufficient).	Prospective observational cohort with 1-year developmental follow-up.	Ferritin ≤34 µg/L indicated suspected brain iron deficiency.	Auditory speech/nonspeech maturation task and mother-versus-stranger voice recognition during active sleep; 5 scalp sites for recognition task.	P2 amplitude/latency and 2 s slow-wave area/difference wave.	Basic auditory P2 maturation was largely preserved. Brain-iron-deficient infants lacked the typical negative slow-wave differentiation between stranger and maternal voices; P2 latency was shorter overall.	No neonatal intervention in the ERP comparison.	Absence of electrophysiological voice recognition was interpreted as impaired recognition memory; developmental follow-up was included.	Ferritin predicted the stranger-minus-mother slow wave specifically at right temporal T4.	Auditory ERP	ERP	No
[34]	Infants (9 and 12 months)	Usable ERP data from 15 infants with iron-deficiency anemia and 19 iron-sufficient infants; longitudinal subgroup 7 + 7.	Cross-sectional and longitudinal developmental comparison.	Infant iron-deficiency anemia defined by low hemoglobin plus ≥2 abnormal iron indices.	Mother-versus-stranger face recognition; 16-channel EEG; frontocentral analyses.	NC amplitude/latency (300–650 ms) and positive slow-wave area (800–1500 ms).	At 9 months, iron-sufficient infants showed the expected mother-related NC and stranger-related PSW pattern, whereas iron-deficient infants showed atypical or reversed differentiation. At 12 months, the iron-deficient group resembled the 9-month iron-sufficient pattern.	Developmental trajectory suggested an approximately 3-month electrophysiological delay.	ERP differences implicated delayed attention allocation and memory updating for familiar versus novel faces.	NC latency was not altered; small sample and residual social/maternal confounding were considered.	Visual ERP	ERP	No
[35]	Adults (20–60 years)	51 adults with newly diagnosed iron-deficiency anemia (46 women, 5 men).	Within-subject pre–post treatment study without a healthy control group.	Hb <11 g/dL with microcytic/hypochromic iron-deficiency profile; 3 months oral ferroglycine sulfate.	Auditory oddball ERPs at Fz/Cz/Pz plus 20 min eyes-closed resting qEEG from 19 electrodes.	N1, P2, N2, P3 amplitudes/latencies; delta through gamma and broadband spectral power.	After treatment, N1 and P2 amplitudes increased, N2 latency shortened, P3 latency shortened at all midline sites, and P3 amplitude increased at Pz. Resting power generally decreased in posterior, temporal, and central regions; gamma was unchanged.	Broad electrophysiological improvement after correction of anemia, although the paper contains an internal wording inconsistency about qEEG direction.	No extensive neuropsychological battery; cognitive improvement was inferred mainly from ERP changes.	Lower hemoglobin was associated with higher pretreatment delta power, suggesting a contribution of cerebral hypoxia.	Mixed EEG + ERP	Mixed EEG/ERP	Yes
[36]	Boys (8–10 years)	34 controls and 18 anemic boys at baseline; paired follow-up included 23 controls and 14 anemic boys.	Controlled pre–post intervention study.	Hb <12 g/dL with reduced red-cell/iron indices; 3–4 mg/kg/day oral iron for 90 days; both groups received albendazole and vitamin C.	Auditory oddball P300 at Cz with button response.	P300 latency and amplitude.	Anemic boys had significantly prolonged P300 latency but similar amplitude. After treatment, latency shortened numerically but remained significantly longer than controls; amplitude remained comparable.	Psychometric recovery was greater than electrophysiological recovery; P300 slowing persisted after 3 months.	Raven and Digit Span scores improved and no longer differed from controls after treatment.	Suggests persistent slowing of stimulus evaluation despite improved hematology and behavioral performance.	Auditory ERP	ERP	Yes
[37]	Children (~10.2 years)	95 children: 51 formerly iron-deficient anemic in infancy and 44 controls.	Long-term follow-up approximately 10 years after treated infantile iron-deficiency anemia.	Infant IDA treated for 6–12 months; current iron deficiency was rare at follow-up.	Continuous old–new visual word-recognition task; 28-channel EEG.	FN400 peak amplitude/latency (300–500 ms) and P300 mean amplitude (500–800 ms).	Controls showed the expected FN400 old–new effect; formerly anemic children showed weak differentiation and significantly delayed FN400. P300 amplitude was reduced at Fz and Cz, with preserved posterior topography.	Abnormalities persisted roughly a decade after infant treatment.	Recognition accuracy was similar, but formerly anemic children were initially slower.	Indicates lasting changes in familiarity/semantic-access and later recognition processing despite near-normal overt performance.	Visual ERP	ERP	Historical treatment
[38]	Infants (~2 months)	162 infants with usable ERP data; ZPP/H analysis: 35 iron deficient and 92 sufficient; ferritin analysis: 28 low and 133 sufficient.	Prospective observational birth cohort.	Fetal–neonatal iron deficiency defined by elevated cord ZPP/H or low cord ferritin; none had IDA.	Mother-versus-stranger voice recognition; 64-channel EEG; frontal-central and parietal-occipital regions.	P2, P750, and late slow wave (LSW).	With elevated ZPP/H, iron-sufficient infants showed the expected frontal-negative/posterior-positive LSW to the stranger voice, whereas iron-deficient infants showed no stimulus-specific LSW differentiation. P2 and P750 topographies were also altered.	Not assessed.	Absent LSW differentiation was interpreted as impaired recognition of the maternal voice.	Effects were much weaker when iron deficiency was defined by ferritin, suggesting ZPP/H may capture functional restriction more sensitively.	Auditory ERP	ERP	No
[39]	Young women (18–27 years)	55 women with low iron stores: 27 biofortified-bean and 28 comparison-bean participants.	Double-blind randomized 128-day feeding trial.	Low ferritin; high prevalence of iron deficiency, with or without anemia; iron-biofortified versus comparison beans.	Five visual tasks: simple reaction time, Go/No-Go, Attention Network, Sternberg memory search, and cued recognition.	P1, N1, P2 amplitudes/latencies; normalized alpha (8–15 Hz) and gamma (30–90 Hz) power.	Biofortification produced larger-magnitude N1 responses across tasks, increased alpha power in simpler attention tasks, and increased gamma power in memory-intensive tasks. ERP latencies and P1 amplitude were not reliably changed.	Changes in ferritin, body iron, and hemoglobin predicted N1 and spectral changes; EEG measures statistically mediated some cognitive benefits.	Improved attentional capture/selection and memory efficiency were linked to neural changes.	Strongest evidence concerned early attentional N1 and task-dependent alpha/gamma recruitment.	Task ERP + spectral EEG	Task ERP/spectral EEG	Yes
[40]	Young adults (~22 years)	28 adults with iron-deficiency anemia and 25 healthy controls.	Prospective case–control study with 3-month pre–post iron treatment.	Clinical iron-deficiency anemia; oral iron correction.	Auditory oddball ERP plus conventional resting wake EEG.	N1/P2/N2/P3 latency and amplitude; qualitative conventional EEG abnormalities.	At baseline, N2 and P3 latencies were prolonged, P2/P3 amplitudes reduced, and 55% had abnormal resting EEG with slowing/paroxysmal activity. Treatment increased P2 and P3 amplitude, but latencies changed little; abnormal EEG prevalence fell to 35%.	Partial, not complete, normalization after 3 months.	Broad neuropsychological impairment improved after treatment but often remained below controls. P3 latency correlated negatively and P3 amplitude positively with IQ/iron measures.	Links electrophysiological abnormalities to both anemia severity and cognitive function.	Mixed EEG + ERP	Mixed EEG/ERP	Yes
[41]	Young adults/undergraduates	108 students; 15 had iron deficiency without anemia and 3 had iron-deficiency anemia.	Observational correlational study in a mostly healthy, non-anemic sample.	Continuous MCH, MCV, ferritin, hemoglobin, and iron indices; subgroup analyses by low/high indices.	Visual task-switching paradigm.	P1, N1, P2, and P3 amplitudes at frontal and posterior-parietal sites.	Lower MCH and MCV consistently predicted altered amplitudes across P1–P3, particularly under switch demands; hemoglobin did not predict ERP outcomes.	Not assessed.	Higher MCH, MCV, and ferritin predicted better Digit Span/working-memory performance.	Suggests subtle red-cell/iron variation may influence processing from early perception through executive updating even without clinical anemia.	Visual ERP	ERP	No
[42]	Girls (8–10 years)	42 girls: 23 anemic and 19 controls.	Cross-sectional controlled study.	Hb <12 g/dL; hematology and iron indices suggested mild anemia/iron restriction.	Auditory oddball P300.	P300 latency and amplitude.	Categorical group differences in P300 did not reach significance, although anemic girls had ~14.5 ms longer latency. Across the full sample, hematocrit correlated inversely with latency; amplitude was not clearly altered.	Not assessed.	Anemic girls showed somewhat poorer psychometric scores, but group differences were not uniformly significant.	Provides weaker evidence than the boys’ treatment study; suggests mild slowing rather than reduced response magnitude.	Auditory ERP	ERP	No
[43]	Children (~8–9 years after infant IDA)	69 children with treated infantile iron-deficiency anemia and 63 comparison children.	Long-term longitudinal follow-up.	IDA during infancy, treated for at least 6 months; comparison infants received preventive iron.	Visual Go/No-Go inhibitory-control task.	N2 amplitude/latency and P300 amplitude.	Formerly anemic children had significantly smaller P300 amplitude and ~22 ms longer N2 latency; N2 amplitude did not differ.	Alterations persisted 8–9 years after treatment.	Accuracy was high but modestly lower in the former IDA group.	Supports lasting inefficiency of inhibitory-control and prefrontal–striatal processing, possibly involving dopamine and myelination.	Visual ERP	ERP	Historical treatment
[44]	Children (8–10 years)	15 iron-deficient, non-anemic children and 15 matched iron-replete controls.	Matched longitudinal supplementation study.	Serum iron <60 µg/dL with depleted stores but no overt anemia; 5 mg/kg/day elemental iron for ~3 months.	Visual verbal Sternberg working-memory task with 3- and 5-digit memory loads.	Late ERP activity, especially P3b/P300-related amplitudes (~580–900 ms).	No group differences at low load. Under 5-digit load, iron-deficient children showed widespread late ERP amplitude abnormalities across frontal, central, temporal, and parietal sites.	After supplementation, ERPs no longer differed from controls.	At high load, deficient children were less accurate and slower; both normalized after treatment.	Demonstrates load-dependent working-memory vulnerability and reversibility with restored iron stores.	Visual ERP	ERP	Yes
[45]	Infants (9 months)	80 infants: 9 deficient prenatally and postnatally, 21 prenatal-only, 20 postnatal-only, and 30 never deficient.	Prospective birth cohort with iron status measured at birth and 9 months.	Prenatal deficiency by cord ferritin/ZPP-H; postnatal deficiency by ≥2 abnormal iron indices.	Two-minute baseline, peek-a-boo, and stranger-approach conditions; 128-channel EEG.	Infant alpha power (6–9 Hz) and frontal asymmetry, ln(F4)–ln(F3).	Only infants deficient at both birth and 9 months showed consistent negative asymmetry, indicating relatively greater right-frontal activation. The effect was present across all conditions and was driven mainly by lower right-frontal alpha.	Not an intervention study; duration/timing of deficiency was the exposure.	Pattern was interpreted as a neurophysiological correlate of altered approach–withdrawal/emotional disposition.	Prenatal-only or postnatal-only deficiency did not show the same asymmetry; combined group was small (*n* = 9).	Frontal asymmetry EEG	Resting/conventional EEG	No
[46]	College-aged women	39 women: 19 iron-deficient non-anemic and 20 matched iron-sufficient controls.	Cross-sectional workload experiment with indirect calorimetry.	Hb ≥12 g/dL and ferritin ≤16 µg/L for IDNA.	Combined Sternberg visual-memory and running-arithmetic task with increasing stimulus complexity; 64-channel EEG.	Normalized alpha, theta, and gamma power relative to eyes-closed baseline.	Iron-deficient women showed greater overall alpha suppression and attenuated theta/gamma recruitment as complexity increased. Ferritin explained 27% of theta-slope and 59% of gamma-slope variance.	Not assessed.	Iron deficiency altered behavioral and metabolic responses to cognitive workload; EEG power helped predict energy expenditure.	Suggests reduced capacity to scale neural recruitment with increasing mental demand.	Task spectral EEG	Task spectral EEG	No
[47]	Infants (3–12 months)	25 infants with iron-deficiency anemia and 25 age-/sex-matched controls; treated infants were later compared with a second age-matched control group.	Prospective pre–post supplementation study.	Hb <11 g/dL plus abnormal iron profile; 5 mg/kg/day ferrous fumarate for 4 months.	≥30 min spontaneous-sleep qEEG from 19 electrodes.	Absolute/relative delta, theta, alpha, and beta power; age- and sex-standardized qEEG Z scores.	At baseline, IDA infants showed excess theta, increased frontocentral delta/theta relative power, and reduced posterior alpha; 64% had an immature qEEG pattern.	After treatment, theta normalized, slow relative power decreased, alpha increased, and 19/25 infants were classified as normal; some alpha deficits persisted.	No primary cognitive task; EEG was interpreted as delayed CNS maturation.	Provides strong evidence of reversible maturational slowing, with incomplete normalization in a minority.	Sleep qEEG	Resting/conventional EEG	Yes
[48]	Young women (20–32 years)	23 healthy non-anemic women: 13 iron deficient and 10 iron sufficient.	Cross-sectional observational study controlling menstrual-cycle phase.	Ferritin <12 µg/L with comparable hemoglobin between groups.	Resting EEG with Mitsar/WinEEG; frontal alpha asymmetry analysis.	Alpha power (8–12 Hz), particularly F3 versus F4.	Iron-deficient women had significantly greater left-midfrontal alpha at F3 and a significant F3–F4 asymmetry not present in controls, consistent with relatively reduced left-frontal activation.	Not assessed.	Accuracy was similar, but iron-deficient women took longer to complete testing and reported lower activity/endurance.	Small sample; asymmetry should not be treated as a direct diagnostic marker of anxiety or depression.	Resting qEEG	Resting/conventional EEG	No
[49]	Adolescents (12–16 years)	Final EEG analysis: 74 participants (41 iron-biofortified millet, 33 comparison); selected from 146 low-ferritin adolescents.	Randomized double-blind 6-month feeding trial.	High prevalence of iron deficiency/IDA; iron-biofortified versus comparison pearl millet.	Five tasks assessing reaction time, Go/No-Go, attentional networks, composite-face processing, and cued recognition; 32-channel EEG.	N1 and P3 amplitudes; alpha and gamma power.	Only N1 amplitude during simple reaction time showed a conventional group-by-time interaction. Broader EEG changes generally favored biofortification, and ferritin/hemoglobin changes predicted task-specific N1, P3, and gamma changes.	Composite EEG changes mediated improvements in attentional capture/selection and memory efficiency.	Ten of 21 behavioral measures improved more in the biofortified group.	Intervention effects were distributed across multivariate neural patterns rather than a single robust EEG endpoint.	Task ERP + spectral EEG	Task ERP/spectral EEG	Yes
[50]	Young women (~24 years)	260 women: 130 with iron-deficiency anemia and 130 healthy controls.	Cross-sectional case–control study.	Hb <12 g/dL and ferritin <15 ng/mL.	Auditory oddball P300 with button response.	P300 latency and amplitude.	P300 latency was significantly prolonged in IDA; amplitude did not differ significantly between groups. Across all participants, latency correlated negatively with hemoglobin; amplitude showed weaker positive relations with iron status.	Not assessed.	Delayed P300 indicated slower attentional allocation, stimulus classification, and working-memory-related processing.	Large sample strengthens evidence for latency slowing in neurologically intact women with IDA.	Auditory ERP	ERP	No
[51]	Infants/young children (~11 and 20 months)	Month 3: 329 usable EEGs (116 iron syrup, 108 micronutrient powder, 105 placebo); month 12: 363 usable EEGs.	Double-blind randomized placebo-controlled supplementation trial with later follow-up.	12.5 mg/day iron syrup or iron-containing multiple micronutrient powder for 3 months versus placebo.	Auditory roving-oddball paradigm assessing novelty and habituation; 32-channel EEG.	Deviant-minus-standard mismatch amplitude (200–400 ms), deviant/standard amplitudes, N2 difference, within-task habituation.	The paradigm elicited a robust novelty response, but neither iron formulation changed mismatch amplitude, N2, stimulus-specific amplitudes, or habituation at month 3 or month 12.	Null electrophysiological intervention effect, including in anemia/iron-deficiency subgroups.	Bayley development was assessed in the parent study; this substudy found no neural habituation benefit.	Large randomized evidence indicates that short-term supplementation did not alter this specific auditory novelty marker.	Auditory ERP	ERP	Yes
[52]	Infants/young children (~11 and 20 months)	Month 3: 412 usable EEGs (138 iron syrup, 139 micronutrient powder, 135 placebo); month 12: 374 usable EEGs.	Double-blind randomized placebo-controlled supplementation trial with follow-up.	12.5 mg/day iron syrup or iron-containing multiple micronutrient powder for 3 months versus placebo.	At least 2 min low-demand resting EEG while viewing Gabor patches; 32 electrodes.	Delta, theta, posterior alpha, central mu alpha, beta; periodic/aperiodic parameterization and peak measures.	Iron syrup increased central mu-alpha power immediately after treatment in both conventional and aperiodic-corrected spectra. No robust effects were found for other bands or for the micronutrient powder.	Mu-alpha difference was absent nine months after supplementation ended.	No task performance outcome in the resting-EEG substudy.	The only corrected immediate effect was a small-to-moderate increase in sensorimotor mu alpha; durability was not demonstrated.	Resting qEEG	Resting/conventional EEG	Yes
[53]	Young women (18–35 years)	42 women: 22 iron-deficient non-anemic and 20 iron-sufficient controls.	Cross-sectional learning experiment.	IDNA defined by depleted ferritin with normal hemoglobin.	Rule-based and information-integration category-learning tasks; 128-channel EEG.	Error-related components including Pe; global field power and neural-efficiency measures.	Iron-deficient women showed markedly reduced Pe amplitudes in both learning systems, persisting even after behavioral performance reached control levels; neural-efficiency trajectories also differed.	Not assessed.	Learning was initially poorer/slower in IDNA, but overt performance could converge despite persistent neural differences.	Suggests altered conscious error awareness and attentional control not fully captured by final accuracy.	Visual ERP	ERP	No
[54]	Infants/young children (6–24 months)	50 children with iron-deficiency anemia or adequate iron status; group sizes not specified in the summary.	Cross-sectional case–control study.	Microcytic hypochromic IDA (Hb <10.5 g/dL plus abnormal red-cell indices) versus non-anemic iron-replete controls.	Flash visual evoked potentials from occipital recordings after monocular stimulation.	N1, P1, and N2 VEP latencies.	N1, P1, and N2 latencies were significantly prolonged for both eyes in the IDA group, indicating delayed visual-pathway conduction.	Not assessed.	No cognitive task; VEPs assessed functional visual-system maturation.	Latency related mainly to anemia severity; ferritin correlated specifically with N2 latency.	VEP	VEP	No
[55]	Infants (9 and 18 months)	At 9 mo: 87 infants (30 fetal–neonatal ID, 20 postnatal ID, 37 sufficient); at 18 mo: 112 (43, 26, and 43).	Prospective longitudinal cohort; partly different samples at each age.	Fetal–neonatal ID by cord ferritin/ZPP-H; postnatal ID by ≥2 abnormal indices at 9 months.	Passive mother-versus-stranger face recognition; 128-channel EEG.	Nc amplitude/latency and late slow wave (LSW) over frontal and temporal regions.	At 9 months, Nc differentiation was preserved across groups, but iron-deficient groups showed altered LSW differentiation/topography. At 18 months, fetal–neonatal ID infants lacked Nc mother–stranger differentiation; postnatal ID showed only right-lateralized differentiation; LSW group effects disappeared.	Developmental expression shifted from late memory-updating abnormalities at 9 months to attention-related Nc abnormalities at 18 months.	Findings indicate timing-specific effects on visual recognition-memory systems.	Only 33 infants had usable data at both ages, limiting strict within-child developmental inference.	Visual ERP	ERP	No
[56]	Infants (7–24 months)	20 infants with iron-deficiency anemia (12 boys, 8 girls).	Within-subject pre–post treatment study without controls.	Laboratory-confirmed IDA; 4 mg/kg/day oral iron sulfate for 12 weeks.	Flash VEP recorded at O1/O2 during pharmacologically assisted sleep.	Primarily N2 VEP latency.	Mean N2 latency shortened from 113.7 to 106.6 ms after treatment, approaching the laboratory age norm.	Significant improvement after anemia resolved.	No cognitive task; result indicates improved visual neural conduction.	No healthy or untreated control and no detailed P100/N1 or amplitude analysis.	VEP	VEP	Yes
[57]	Adults	15 predialysis CKD patients, 15 hemodialysis patients, and 30 healthy controls.	Controlled pre–post erythropoietin treatment study.	Severe anemia secondary to CKD; relatively high ferritin; erythropoietin 100 IU/kg twice weekly for 6 weeks.	Auditory oddball P300.	P300 latency and amplitude.	Both CKD groups had markedly prolonged latency and reduced amplitude. Erythropoietin significantly shortened latency in both groups and increased amplitude significantly in dialysis patients, but values remained abnormal versus controls.	Partial improvement with increased hemoglobin, independent of major renal-function changes.	P300 served as an objective marker of cognitive dysfunction related to anemia/hypoxia.	Not a pure iron-deficiency study; mechanism concerns correction of anemia and oxygen delivery.	Auditory ERP	ERP	Yes
[58]	Adults (18–65 years)	52 adults with anemia: 55.8% IDA, 28.8% megaloblastic anemia, 15.4% anemia of chronic disease.	Prospective observational conventional-EEG study.	Mild, moderate, or severe anemia of mixed etiology.	Resting eyes-closed/open EEG with hyperventilation and photic activation.	Background rhythm frequency/amplitude/reactivity, diffuse/focal slowing, paroxysmal or sharp activity.	EEG abnormalities increased with anemia severity and commonly included theta/delta slowing and reduced alpha frequency/amplitude. Abnormal EEG prevalence was 41.2% in mild, 70.8% in moderate, and 81.8% in severe anemia.	Not assessed.	No formal cognitive ERP task.	Mixed anemia etiologies; megaloblastic anemia had the highest abnormal-EEG prevalence, so findings are not specific to iron deficiency.	Conventional EEG	Resting/conventional EEG	No
[59]	Late-adolescent women (18–19 years)	74 female medical students: 32 anemic and 42 non-anemic controls.	Cross-sectional controlled study.	Anemia defined solely by Hb <12 g/dL; iron deficiency was not biochemically confirmed.	Auditory oddball P300.	P300 latency and amplitude.	Anemic participants had substantially prolonged P300 latency (332.5 vs. 290.6 ms) and reduced amplitude (9.1 vs. 11.7 µV).	Not assessed.	Findings indicate slower stimulus evaluation and weaker attentional-resource allocation.	Because classification used hemoglobin alone, results cannot be attributed specifically to iron deficiency.	Auditory ERP	ERP	No
[60]	Children (7–12 years)	70 children with IDA randomized to active (35) or placebo (35), plus 30 healthy controls; final ERP analysis 33 active, 34 placebo, 30 controls.	Double-blind placebo-controlled 3-month pilot trial.	Hb <120 g/L plus low ferritin or elevated free erythrocyte protoporphyrin; active beverage provided 10 mg iron plus vitamin C, malic acid, and folate.	Auditory oddball with rare-tone counting; vertex recording.	N1, P2, N2, P3 latencies; N2/P3 amplitudes; waveform morphology.	Both anemic groups had ~29 ms prolonged P3 latency and more abnormal waveforms at baseline. Active supplementation significantly shortened P3 latency and improved waveform morphology; amplitudes were unchanged.	Treated children no longer differed from controls, whereas placebo children retained abnormalities; some nonspecific latency shortening occurred in placebo.	IQ improved in the active group and differed from placebo after treatment.	Supports treatment responsiveness of P300 timing/morphology more strongly than amplitude.	Auditory ERP	ERP	Yes

**Table 3:** Risk of bias assessment (ROBINS-I).

Study	Bias Due to Confounding	Bias in Selection of Participants	Bias in Classification or Measurement of Exposure	Bias Due to Deviations from the Exposure	Bias Due to Missing Data	Bias in Measurement of Outcomes	Bias in Selection of the Reported Result
[31]	Serious	Moderate	Moderate	Moderate	Moderate	Moderate	Serious
[32]	Serious	Moderate	Moderate	Not applicable/Low	Low risk, with some uncertainty	Moderate	Moderate
[33]	Serious	Serious	Low	Moderate	Serious	Moderate	Moderate
[34]	Serious	Moderate	Moderate	Low risk for the neonatal EEG outcome	Moderate	Low	Moderate
[35]	Critical	Serious	Low	Moderate	Serious	Moderate	Serious
[36]	Serious	Low	Low	Moderate	Serious	Moderate	Moderate
[37]	Serious	Moderate	Low	Moderate	Serious	Moderate	Moderate
[38]	Serious	Moderate	Moderate	Moderate	Serious	Low	Serious
[40]	Serious	Moderate	Low	Serious	Moderate to Serious	Serious	Serious
[41]	Serious	Moderate	Low	Not applicable/Low	Moderate	Moderate	Serious
[42]	Serious	Moderate	Moderate	Not applicable/Low	Low	Moderate	Serious
[43]	Serious	Serious	Low	Moderate	Serious	Moderate	Moderate
[44]	Serious	Serious	Low	Moderate	Serious	Moderate	Moderate
[45]	Serious	Low	Moderate	Not applicable/Low	Serious	Low	Moderate
[46]	Serious	Moderate	Low	Low	Low	Moderate	Serious
[47]	Serious	Moderate	Low	Moderate	Low	Moderate	Moderate
[48]	Serious	Moderate	Low	Not applicable/Low	Low	Moderate	Serious
[50]	Serious	Moderate	Low	Not applicable/Low	Low	Moderate	Moderate
[53]	Serious	Moderate	Low	Low	Moderate	Moderate	Moderate
[54]	Serious	Moderate	Low	Low	Low	Moderate	Moderate
[55]	Serious	Serious	Moderate	Moderate	Serious	Moderate	Serious
[56]	Critical	Moderate	Low	Moderate	Low	Moderate	Serious
[57]	Serious	Low	Low	Moderate	Moderate	Moderate	Moderate
[58]	Serious	Serious	Moderate	Not applicable/Low	Moderate	Moderate	Serious
[59]	Serious	Serious	Moderate for anemia; critical for iron deficiency	Not applicable/Low	Moderate	Serious	Moderate

**Table 4:** Risk of bias assessment (RoB-2).

Study	Bias Arising from the Randomization Process	Bias Due to Deviations from Intended Interventions	Bias Due to Missing Outcome Data	Bias in Measurement of the Outcome	Bias in Selection of the Reported Result
[39]	Some concerns	Low risk	Some concerns	Low risk	High risk
[49]	Some concerns	Some concerns	Some concerns	Low risk	Some concerns for direct randomized comparisons; high risk for mediation results
[51]	Low risk	Low risk	Some concerns	Low risk	Low risk
[52]	Some concerns	Low risk	Low risk for the month-3 result	Low risk	Some concerns
[60]	Some concerns	Low risk	Some concerns	Low risk for P300 latency; some concerns for waveform abnormality	High risk

## Data Availability

No new data were created or analyzed in this study. Data sharing is not applicable to this article.

## Supplementary Materials

The following supporting information can be downloaded at: https://www.mdpi.com/article/10.3390/nu18182955/s1, Table S1: Different search strategies for different databases.
