# Supplementary material for: Iron Deficiency and Human Brain Electrophysiology Across the Lifespan: A Systematic and Mechanistic Review of Resting EEG, Event-Related Potentials, and Treatment Responsiveness
Source: Nutrients. 2026 Sep 9;18(18):2955. doi: 10.3390/nu18182955 (PMC13610947; doi:10.3390/nu18182955)
Supplement: Supplementary file 1 [file nutrients-18-02955-s001.zip › nutrients-4485944-supplementary.pdf]

**Table S1.** Different search strategies for different databases.

| Database / information source | Platform | Date searched | Coverage | Exact search strategy | Limits / filters | Records retrieved / screened |
|---|---|---|---|---|---|---|
| PubMed/MEDLINE | PubMed | 1 July 2026 | Inception to 1 July 2026 | (("iron deficiency"[Title/Abstract] OR "iron deficient"[Title/Abstract] OR "iron depletion"[Title/Abstract] OR "depleted iron stores"[Title/Abstract] OR "low iron"[Title/Abstract] OR "low ferritin"[Title/Abstract] OR "iron-deficiency anemia"[Title/Abstract] OR "iron deficiency anaemia"[Title/Abstract] OR IDA[Title/Abstract]) AND (EEG[Title/Abstract] OR electroencephalography[Title/Abstract] OR electroencephalogram[Title/Abstract] OR qEEG[Title/Abstract] OR "quantitative EEG"[Title/Abstract] OR "brain electrical activity"[Title/Abstract] OR "event-related potential"[Title/Abstract] OR "event related potential"[Title/Abstract] OR ERP[Title/Abstract] OR "evoked potential"[Title/Abstract] OR P300[Title/Abstract] OR P3[Title/Abstract] OR P1[Title/Abstract] OR N1[Title/Abstract] OR N100[Title/Abstract] OR P2[Title/Abstract] OR P200[Title/Abstract] OR N2[Title/Abstract] OR N200[Title/Abstract] OR "slow | None. No restrictions by age, sex, geographical location, or publication year. | 21 |

| Database / information source | Platform | Date searched | Coverage | Exact search strategy | Limits / filters | Records retrieved / screened |
|---|---|---|---|---|---|---|
| | | | | wave"[Title/Abstract] OR FN400[Title/Abstract] OR "spectral power"[Title/Abstract] OR "brain oscillations"[Title/Abstract] OR "alpha asymmetry"[Title/Abstract])) | | |
| Scopus | Elsevier Scopus | 1 July 2026 | Inception to 1 July 2026 | TITLE-ABS-KEY("iron deficiency" OR "iron deficient" OR "iron depletion" OR "depleted iron stores" OR "low iron" OR "low ferritin" OR "iron-deficiency anemia" OR "iron deficiency anaemia" OR IDA) AND TITLE-ABS-KEY(EEG OR electroencephalography OR electroencephalogram OR qEEG OR "quantitative EEG" OR "brain electrical activity" OR "event-related potential" OR "event related potential" OR ERP OR "evoked potential" OR P300 OR P3 OR P1 OR N1 OR N100 OR P2 OR P200 OR N2 OR N200 OR "slow wave" OR FN400 OR "spectral power" OR "brain oscillations" OR "alpha asymmetry") | None. No restrictions by age, sex, geographical location, or publication year. | 24 |
| Web of Science Core Collection | Clarivate Web of Science | 1 July 2026 | Inception to 1 July 2026 | TS=("iron deficiency" OR "iron deficient" OR "iron depletion" OR "depleted iron stores" OR "low iron" OR "low ferritin" OR "iron-deficiency anemia" OR "iron deficiency anaemia" OR IDA) AND TS=(EEG OR electroencephalography OR electroencephalogram OR qEEG OR "quantitative EEG" OR "brain electrical activity" OR "event-related potential" OR "event related potential" OR ERP | None. All document years searched; no restrictions by age, sex, or geographical location. | 18 |

| Database / information source | Platform | Date searched | Coverage | Exact search strategy | Limits / filters | Records retrieved / screened |
|---|---|---|---|---|---|---|
| | | | | OR "evoked potential" OR P300 OR P3 OR P1 OR N1 OR N100 OR P2 OR P200 OR N2 OR N200 OR "slow wave" OR FN400 OR "spectral power" OR "brain oscillations" OR "alpha asymmetry") | | |
| Embase | Embase.com (Elsevier) | 1 July 2026 | Inception to 1 July 2026 | ('iron deficiency':ti,ab,kw OR 'iron deficient':ti,ab,kw OR 'iron depletion':ti,ab,kw OR 'depleted iron stores':ti,ab,kw OR 'low iron':ti,ab,kw OR 'low ferritin':ti,ab,kw OR 'iron-deficiency anemia':ti,ab,kw OR 'iron deficiency anaemia':ti,ab,kw OR ida:ti,ab,kw) AND (eeg:ti,ab,kw OR electroencephalography:ti,ab,kw OR electroencephalogram:ti,ab,kw OR qeeg:ti,ab,kw OR 'quantitative EEG':ti,ab,kw OR 'brain electrical activity':ti,ab,kw OR 'event-related potential':ti,ab,kw OR 'event related potential':ti,ab,kw OR erp:ti,ab,kw OR 'evoked potential':ti,ab,kw OR p300:ti,ab,kw OR p3:ti,ab,kw OR p1:ti,ab,kw OR n1:ti,ab,kw OR n100:ti,ab,kw OR p2:ti,ab,kw OR p200:ti,ab,kw OR n2:ti,ab,kw OR n200:ti,ab,kw OR 'slow wave':ti,ab,kw OR fn400:ti,ab,kw OR 'spectral power':ti,ab,kw OR 'brain oscillations':ti,ab,kw OR 'alpha asymmetry':ti,ab,kw) | None. No restrictions by age, sex, geographical location, or publication year. | 17 |
| PsycINFO | APA PsycINFO via EBSCOhost | 1 July 2026 | Inception to 1 July 2026 | (TI ("iron deficiency" OR "iron deficient" OR "iron depletion" OR | None. No restrictions by age, sex, geographical | 7 |

| Database / information source | Platform | Date searched | Coverage | Exact search strategy | Limits / filters | Records retrieved / screened |
|---|---|---|---|---|---|---|
| | | | | "depleted iron stores" OR "low iron" OR "low ferritin" OR "iron-deficiency anemia" OR "iron deficiency anaemia" OR IDA) OR AB ("iron deficiency" OR "iron deficient" OR "iron depletion" OR "depleted iron stores" OR "low iron" OR "low ferritin" OR "iron-deficiency anemia" OR "iron deficiency anaemia" OR IDA)) AND (TI (EEG OR electroencephalography OR electroencephalogram OR qEEG OR "quantitative EEG" OR "brain electrical activity" OR "event-related potential" OR "event related potential" OR ERP OR "evoked potential" OR P300 OR P3 OR P1 OR N1 OR N100 OR P2 OR P200 OR N2 OR N200 OR "slow wave" OR FN400 OR "spectral power" OR "brain oscillations" OR "alpha asymmetry") OR AB (EEG OR electroencephalography OR electroencephalogram OR qEEG OR "quantitative EEG" OR "brain electrical activity" OR "event-related potential" OR "event related potential" OR ERP OR "evoked potential" OR P300 OR P3 OR P1 OR N1 OR N100 OR P2 OR P200 OR N2 OR N200 OR "slow wave" OR FN400 OR "spectral power" OR "brain oscillations" OR "alpha asymmetry")) | location, or publication year. | |
| Google Scholar | Google Scholar | 1 July 2026 | All available years to 1 July 2026 | Searches conducted separately as: "iron deficiency" EEG; "iron deficiency anemia" EEG; "iron deficiency" "event- | Google Scholar was used as a supplementary source. | 14 |

| Database / information source | Platform | Date searched | Coverage | Exact search strategy | Limits / filters | Records retrieved / screened |
|---|---|---|---|---|---|---|
| | | | | related potential"; "iron deficiency anemia" "event-related potential"; "iron deficiency" P300; "low ferritin" EEG | Results were sorted using the default relevance ranking. The first 200 results from each query were screened, or all available results when fewer than 200 were returned. Citations already retrieved from the bibliographic databases and duplicate citations across Google Scholar queries were excluded from further assessment. | |
